# Supplementary material for: Gene-level analysis of rare variants in 379,066 whole exome sequences identifies an association of GIGYF1 loss of function with type 2 diabetes
Source: Sci Rep. 2021 Nov 3;11:21565. doi: 10.1038/s41598-021-99091-5 (PMC8566487; doi:10.1038/s41598-021-99091-5)
Supplement: Supplementary file 1 — Supplementary Information 1. [file 41598_2021_99091_MOESM1_ESM.docx]

# Gene-level analysis of rare variants in 379,066 whole exome sequences identifies an association of *GIGYF1* loss of function with type 2 diabetes

Aimee M. Deaton^1*^, Margaret M. Parker^1^, Lucas D. Ward^1^, Alexander O. Flynn-Carroll^1^, Lucas BonDurant^1^, Gregory Hinkle^1^, Parsa Akbari^2^, Luca A. Lotta^2^, Regeneron Genetics Center^2**^, DiscovEHR Collaboration^3**^, Aris Baras^2^ and Paul Nioi^1^

^1^Alnylam Pharmaceuticals, Cambridge, MA

^2^Regeneron Genetics Center, Regeneron Pharmaceuticals, Tarrytown, NY

^3^Geisinger Health System, Danville, PA

* Correspondence to [adeaton@alnylam.com](mailto:adeaton@alnylam.com)

** For full author and contribution list see Supplementary Information.

# Supplementary Information

# Supplementary Figures

## Supplementary Figure 1: QQ plots for gene-based burden tests.

QQ plots stratified by the number of carriers measured are shown for A) glucose and B) HbA1c. QQ plots stratified by the minimum number of expected case carriers is shown for C) T2D diagnosis. The genomic inflation factor lambda at different thresholds is displayed.


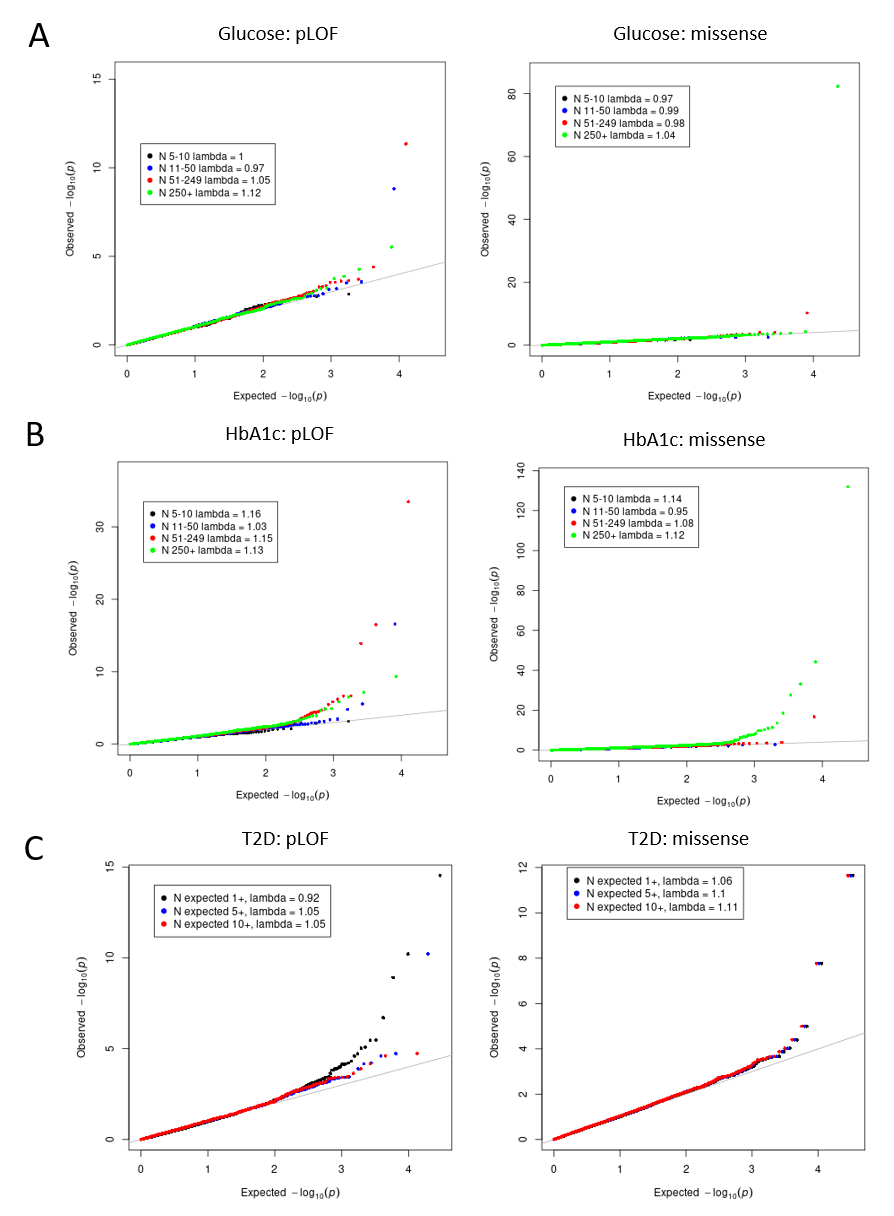


## Supplementary Figure 2: Associations with glucose and HbA1c levels from primary care data

Forest plot showing associations with glucose and HbA1c levels as measured in primary care (GP) data. Effect size in standard deviations is shown along with the 95% confidence interval. The threshold for significance is p ≤ 0.004 correcting for the number of genes and variant sets tested.


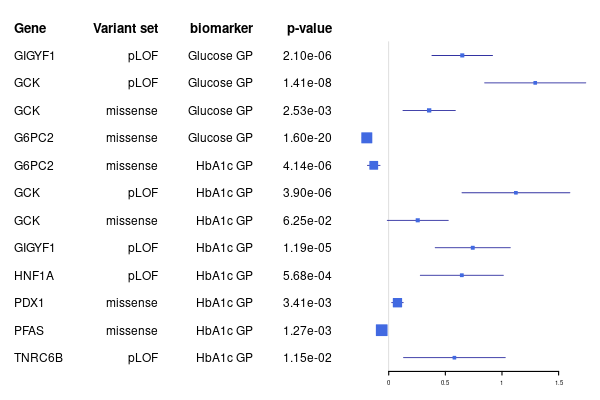


## Supplementary Figure 3: Leave one variant out analysis

Burden tests were run for significant variant sets of interest leaving out one variant at a time. Negative log10 of the p-value for each test is shown relative to the genomic position of the excluded variant. A) Glucose associations B) HbA1c associations C) T2D associations. pLOF variant sets are labeled as “lof.hc” and damaging missense variant sets as “cadd.25”.


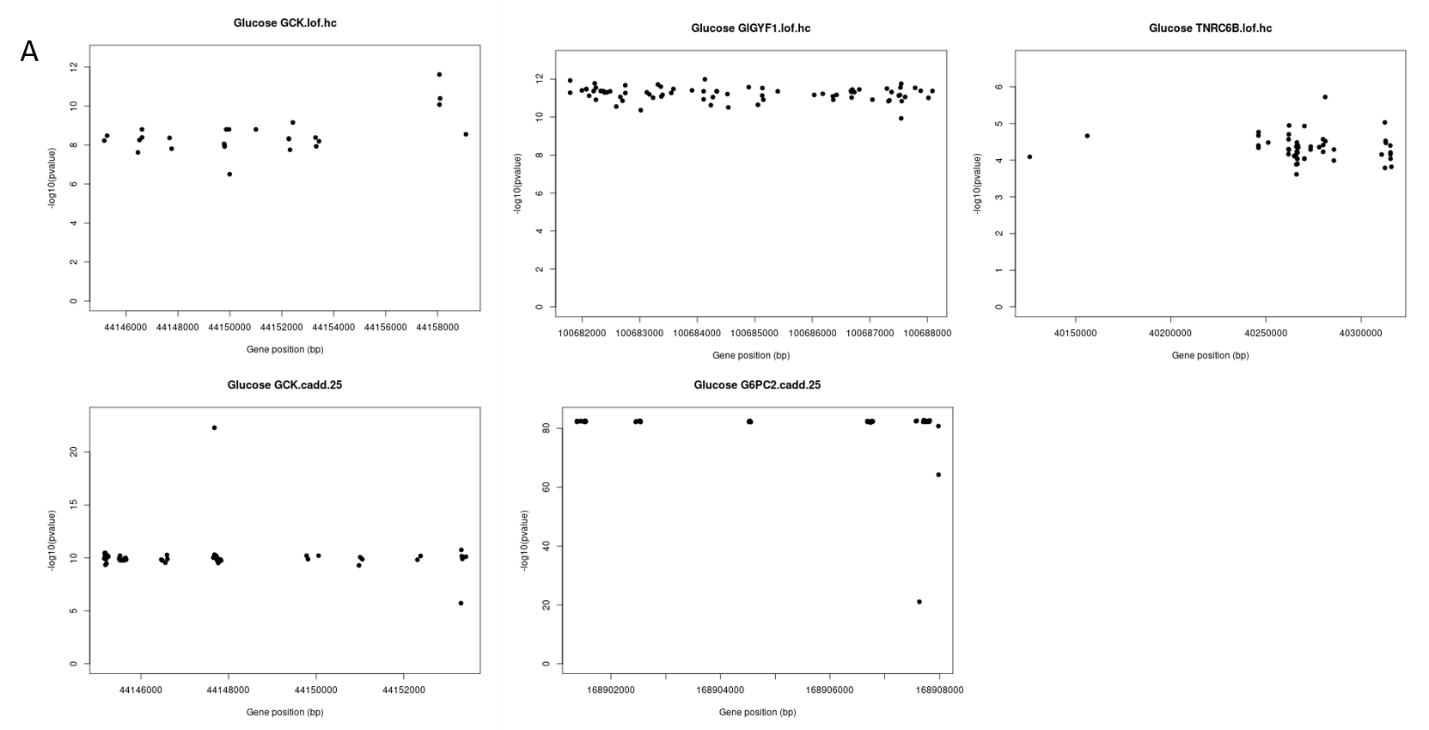


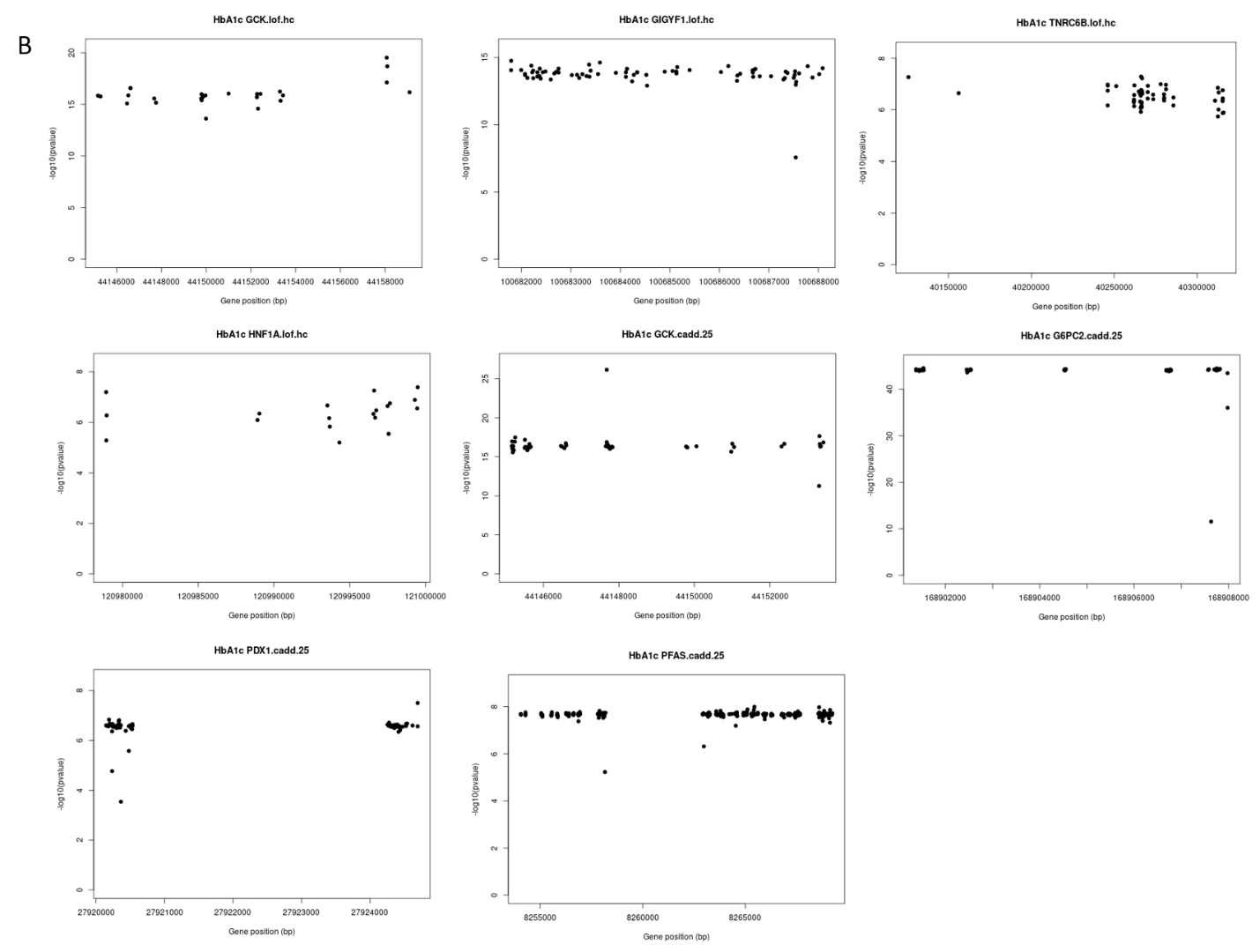

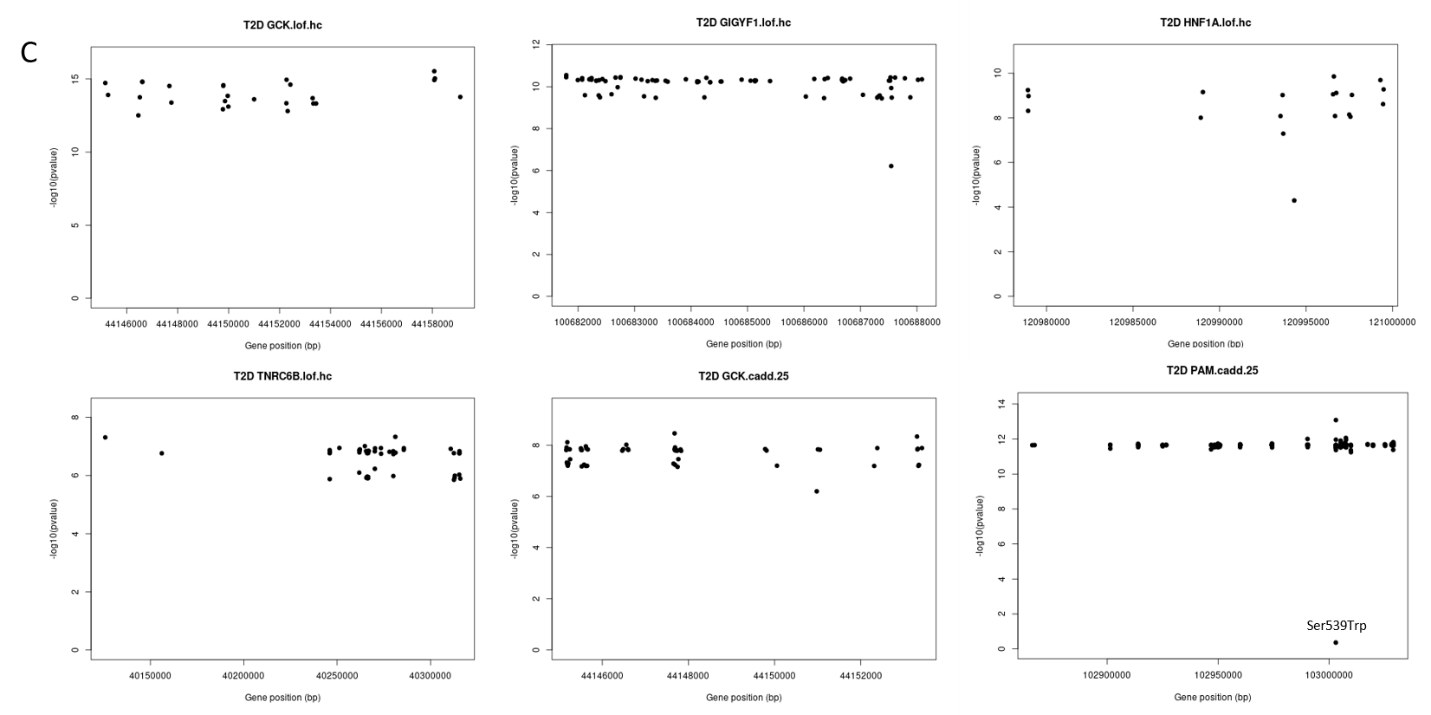


## Supplementary Figure 4: Location of variants of interest in *GIGYF1, PFAS* and *TNRC6B*

The locations of individual variants making up the variant sets are plotted with respect to Ensembl transcripts. Selected variants are shown as a separate track. A) *GIGYF1* pLOF variants, 7:100687545:CA:C shown separately. B) *PFAS* damaging missense variants, 17:8258163:G:A (Glu434Lys) and 17:8262979:G:A (Ala466Thr) are shown separately. C) *TNRC6B* pLOF variants, 22:40270205:G:T and 22:40281161:G:T shown separately.


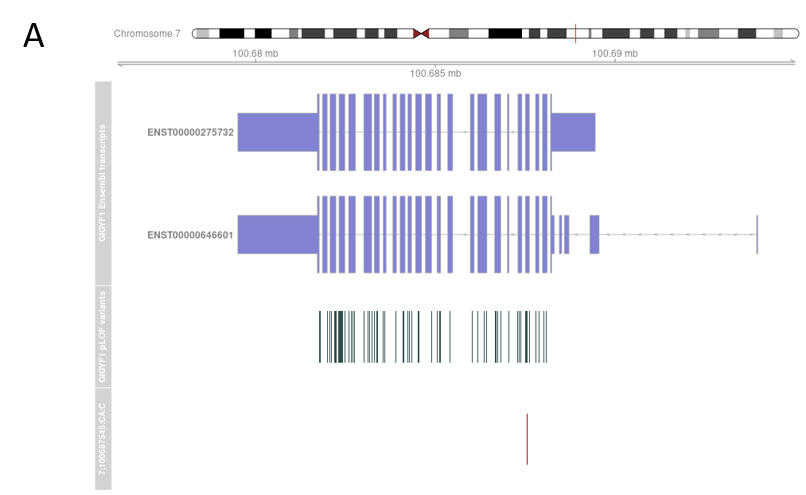

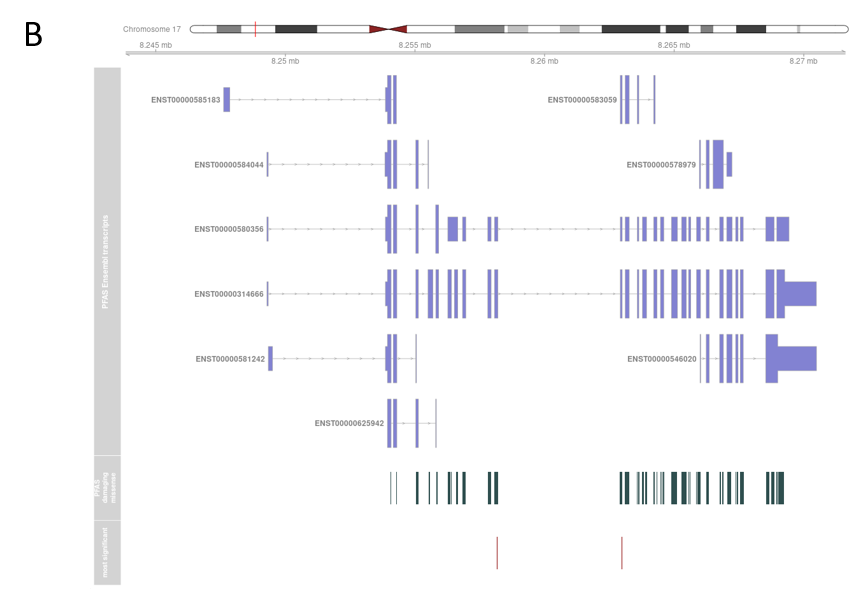


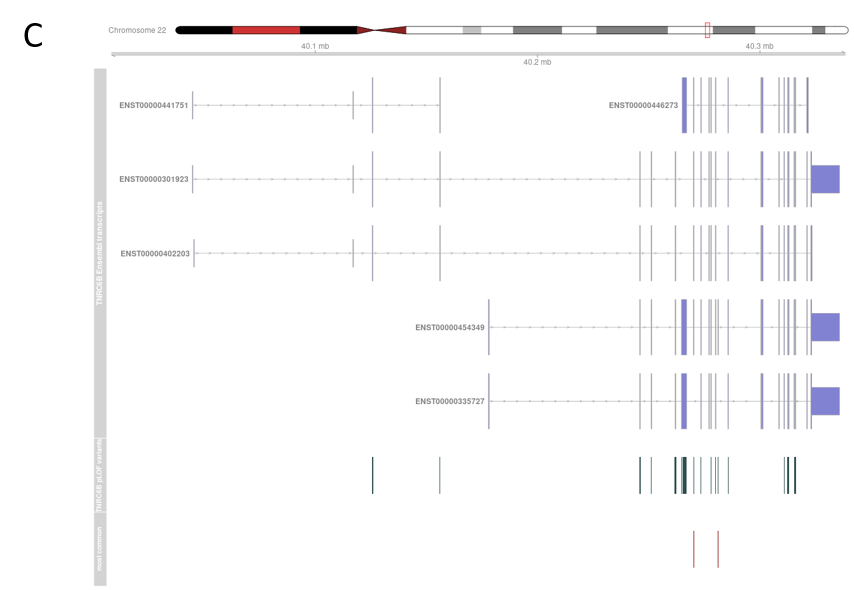


## Supplementary Figure 5: Association of drug target genes with HbA1c and T2D

Associations with HbA1c and T2D for variants in 8 genes encoding T2D drug targets (agonist targets: *GLP1R, IGF1R, PPARG, INSR*; antagonist targets: *SLC5A2, DPP4, KCNJ11, ABCC8*). The red line indicates the significance threshold (p < 0.003 correcting for 15 variant sets tested) and the blue line indicates nominal significance of p < 0.05.


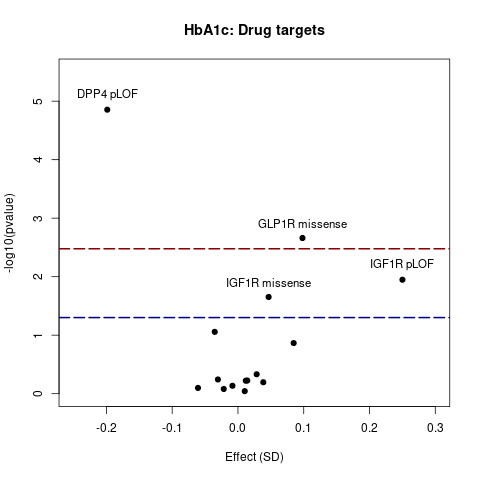

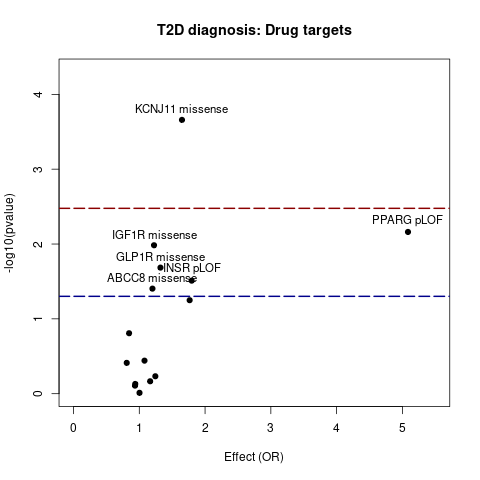


## Supplementary Figure 6: Associations for genes near T2D GWAS loci

All genes in our study are colored grey and the two genes nearest T2D GWAS loci [1] are colored black. Red genes show significant associations with T2D or HbA1c in our study (p < 2.41 x10^-5^). Genes shown twice have two variant sets (pLOF and missense) associating with the trait.


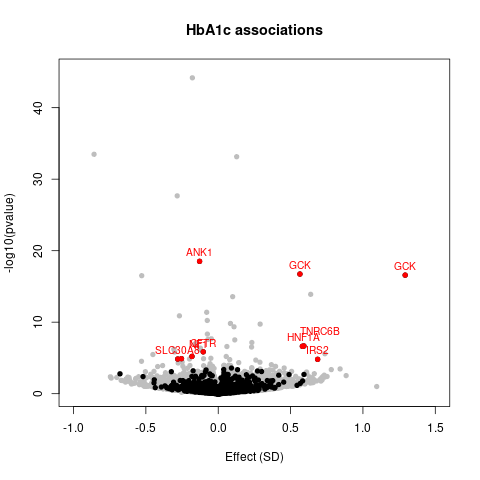

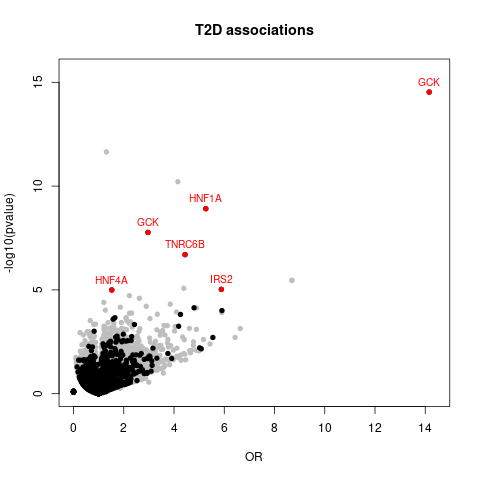


# Supplementary Tables

## Supplementary Table 1: Numbers of pLOF and damaging missense variants in UKBB

Information for rare variants (MAF ≤ 1%) in each subpopulation of UKBB. CADD; combined annotation dependent depletion score.

| **Population** | **N pLOF** | **N pLOF singletons** | **N genes with pLOF** | **N missense (CADD ≥ 25)** | **N missense (CADD ≥ 25) singletons** | **N genes with missense (CADD ≥ 25)** | **N total variant sets (pLOF+missense)** |
| --- | --- | --- | --- | --- | --- | --- | --- |
| All | 726,422 | 425,081 | 16,477 | 2,139,471 | 1,060,539 | 17,312 | 33,789 |
| European | 574,950 | 338,916 | 16,348 | 1,691,784 | 838,024 | 17,222 | 33,570 |
| African | 38,888 | 27,955 | 8,082 | 174,550 | 120,895 | 14,645 | 22,727 |
| South Asian | 47,511 | 33,045 | 8,988 | 212,846 | 140,152 | 15,169 | 24,157 |
| East Asian | 9,336 | 6,841 | 2,987 | 53,486 | 39,008 | 9,909 | 12,896 |

## Supplementary Table 2: Individual variants in genes associating with glucose, HbA1c or T2D diagnosis

Supplied as an excel file. pLOF variants are labeled as “lof.hc” and damaging missense variants are labeled as “cadd.25”.

## Supplementary Table 3: T2D associations for participants recruited in England

| **gene** | **Variant set** | **title** | **population** | **pvalue** | **OR** | **95% CI -** | **95% CI +** | **n cases** | **n carrier** | **n carrier cases** | **n expected** |
| --- | --- | --- | --- | --- | --- | --- | --- | --- | --- | --- | --- |
| GCK | missense CADD ≥ 25 | T2D | English recruited | 1.38x10^-8^ | 3.11 | 2.1 | 4.6 | 22828 | 177 | 32 | 12.59 |
| GCK | pLOF | T2D | English recruited | 1.51x10^-14^ | 14.63 | 7.38 | 29 | 22828 | 37 | 18 | 2.63 |
| GIGYF1 | pLOF | T2D | English recruited | 1.24x10^-10^ | 4.33 | 2.77 | 6.76 | 22828 | 111 | 27 | 7.89 |
| HNF1A | pLOF | T2D | English recruited | 4.05x10^-10^ | 5.64 | 3.28 | 9.7 | 22828 | 67 | 20 | 4.77 |
| PAM | missense CADD ≥ 25 | T2D | English recruited | 6.34x10^-13^ | 1.33 | 1.23 | 1.44 | 22828 | 8242 | 751 | 586.21 |
| TNRC6B | pLOF | T2D | English recruited | 6.30x10^-7^ | 4.39 | 2.45 | 7.85 | 22828 | 63 | 16 | 4.48 |

## Supplementary Table 4: T2D associations adjusting for follow-up duration

Gene-level associations with T2D diagnosis were performed on the variant sets significant in the primary analysis adjusting for follow-up duration.

| **gene** | **Variant set** | **title** | **pvalue** | **OR** | **95% CI-** | **95% CI+** | **n carrier cases** | **n expected** |
| --- | --- | --- | --- | --- | --- | --- | --- | --- |
| GCK | pLOF | T2D | 8.99x10^-16^ | 14.36 | 7.50 | 27.48 | 19 | 2.71 |
| PAM | missense CADD>25 | T2D | 3.30x10^-12^ | 1.30 | 1.21 | 1.40 | 801 | 634.85 |
| GIGYF1 | pLOF | T2D | 4.91x10^-12^ | 4.43 | 2.90 | 6.75 | 29 | 8.89 |
| HNF1A | pLOF | T2D | 6.77x10^-10^ | 5.26 | 3.11 | 8.92 | 20 | 4.95 |
| GCK | missense CADD>25 | T2D | 4.58x10^-8^ | 2.84 | 1.95 | 4.13 | 34 | 13.71 |
| TNRC6B | pLOF | T2D | 1.71x10^-7^ | 4.40 | 2.53 | 7.68 | 17 | 4.82 |

## Supplementary Table 5: Glucose and HbA1c associations in men

Results for variant sets identified in the primary analysis are shown, no additional significant associations were found.

| **gene** | **Variant set** | **title** | **pvalue** | **effect (SD)** | **95% CI-** | **95% CI+** | **N carrier measured** | **N** | **significant in sex-stratified** |
| --- | --- | --- | --- | --- | --- | --- | --- | --- | --- |
| G6PC2 | missense | glucose | 5.68E-34 | -0.24 | -0.28 | -0.21 | 2418 | 167178 | Y |
| GIGYF1 | pLOF | glucose | 7.13E-07 | 0.6 | 0.36 | 0.83 | 67 | 167178 | Y |
| GCK | missense | glucose | 0.0001 | 0.43 | 0.21 | 0.65 | 77 | 167178 | N |
| GCK | pLOF | glucose | 0.0178 | 0.58 | 0.1 | 1.06 | 16 | 167178 | N |
| PIEZO1 | missense | HbA1c | 6.44E-54 | -0.14 | -0.16 | -0.12 | 12320 | 167178 | Y |
| G6PC2 | missense | HbA1c | 3.01E-21 | -0.18 | -0.22 | -0.14 | 2580 | 167178 | Y |
| RHAG | pLOF | HbA1c | 1.02E-20 | -0.99 | -1.19 | -0.78 | 82 | 167178 | Y |
| AMPD3 | missense | HbA1c | 5.24E-16 | 0.13 | 0.1 | 0.16 | 3815 | 167178 | Y |
| PFKM | missense | HbA1c | 3.34E-14 | -0.29 | -0.37 | -0.22 | 622 | 167178 | Y |
| EPB41 | pLOF | HbA1c | 1.88E-12 | -0.65 | -0.83 | -0.47 | 107 | 167178 | Y |
| GIGYF1 | pLOF | HbA1c | 1.91E-11 | 0.74 | 0.53 | 0.96 | 75 | 167178 | Y |
| GCK | missense | HbA1c | 1.18E-07 | 0.53 | 0.33 | 0.72 | 92 | 167178 | Y |
| ANK1 | missense | HbA1c | 4.32E-07 | -0.11 | -0.15 | -0.07 | 1980 | 167178 | Y |
| PFKL | missense | HbA1c | 7.89E-07 | 0.1 | 0.06 | 0.13 | 2441 | 167178 | Y |
| GCK | pLOF | HbA1c | 1.20E-06 | 1.07 | 0.64 | 1.5 | 19 | 167178 | N |
| PDX1 | missense | HbA1c | 9.00E-06 | 0.08 | 0.04 | 0.11 | 3063 | 167178 | N |
| PLD1 | missense | HbA1c | 2.14E-05 | 0.08 | 0.05 | 0.12 | 2410 | 167178 | N |
| PLA2G12A | missense | HbA1c | 2.46E-05 | -0.07 | -0.11 | -0.04 | 3142 | 167178 | N |
| TFR2 | missense | HbA1c | 3.64E-05 | -0.15 | -0.22 | -0.08 | 708 | 167178 | N |
| BLVRB | missense | HbA1c | 4.24E-05 | -0.08 | -0.11 | -0.04 | 2609 | 167178 | N |
| CARHSP1 | missense | HbA1c | 6.93E-05 | -0.15 | -0.22 | -0.08 | 644 | 167178 | N |
| PTPRH | pLOF | HbA1c | 7.12E-05 | 0.1 | 0.05 | 0.16 | 1339 | 167178 | N |
| EPB42 | pLOF | HbA1c | 1.09E-04 | -0.35 | -0.53 | -0.17 | 112 | 167178 | N |
| AXL | missense | HbA1c | 1.28E-04 | -0.07 | -0.1 | -0.03 | 3064 | 167178 | N |
| TNFRSF13B | missense | HbA1c | 2.33E-04 | -0.07 | -0.11 | -0.03 | 2519 | 167178 | N |
| LCAT | missense | HbA1c | 2.37E-04 | -0.22 | -0.33 | -0.1 | 265 | 167178 | N |
| APEH | missense | HbA1c | 0.00065 | 0.23 | 0.1 | 0.36 | 208 | 167178 | N |
| HK1 | missense | HbA1c | 0.0007 | -0.15 | -0.24 | -0.06 | 467 | 167178 | N |
| PLD1 | pLOF | HbA1c | 0.0011 | 0.22 | 0.09 | 0.35 | 208 | 167178 | N |
| PFAS | missense | HbA1c | 0.0019 | -0.04 | -0.06 | -0.01 | 5792 | 167178 | N |
| APOB | pLOF | HbA1c | 0.0022 | 0.2 | 0.07 | 0.33 | 206 | 167178 | N |
| SLC4A1 | missense | HbA1c | 0.0043 | -0.13 | -0.21 | -0.04 | 470 | 167178 | N |
| TNRC6B | pLOF | HbA1c | 0.0079 | 0.42 | 0.11 | 0.74 | 36 | 167178 | N |
| TMC8 | missense | HbA1c | 0.046 | 0.06 | 0 | 0.13 | 914 | 167178 | N |
| HNF1A | pLOF | HbA1c | 0.087 | 0.29 | -0.04 | 0.61 | 33 | 167178 | N |

## Supplementary Table 6: Glucose and HbA1c associations in women

Results for variant sets identified in the primary analysis are shown, no additional significant associations were found.

| **gene** | **variant set** | **title** | **pvalue** | **effect (SD)** | **95% CI-** | **95% CI+** | **N carrier measured** | **N** | **significant in sex-stratified** |
| --- | --- | --- | --- | --- | --- | --- | --- | --- | --- |
| G6PC2 | missense | glucose | 1.03E-51 | -0.29 | -0.32 | -0.25 | 2708 | 196428 | Y |
| GCK | pLOF | glucose | 3.73E-10 | 1.4 | 0.96 | 1.84 | 19 | 196428 | Y |
| GCK | missense | glucose | 7.62E-08 | 0.53 | 0.34 | 0.73 | 96 | 196428 | Y |
| GIGYF1 | pLOF | glucose | 1.49E-06 | 0.64 | 0.38 | 0.9 | 54 | 196428 | N |
| PIEZO1 | missense | HbA1c | 1.26E-82 | -0.15 | -0.17 | -0.14 | 14380 | 196428 | Y |
| G6PC2 | missense | HbA1c | 4.22E-25 | -0.18 | -0.21 | -0.14 | 2989 | 196428 | Y |
| AMPD3 | missense | HbA1c | 3.14E-19 | 0.13 | 0.1 | 0.15 | 4434 | 196428 | Y |
| PFKM | missense | HbA1c | 1.37E-15 | -0.27 | -0.34 | -0.21 | 730 | 196428 | Y |
| RHAG | pLOF | HbA1c | 2.84E-15 | -0.74 | -0.92 | -0.56 | 97 | 196428 | Y |
| ANK1 | missense | HbA1c | 3.29E-14 | -0.15 | -0.18 | -0.11 | 2357 | 196428 | Y |
| GCK | pLOF | HbA1c | 5.86E-14 | 1.59 | 1.18 | 2.01 | 19 | 196428 | Y |
| GCK | missense | HbA1c | 1.12E-11 | 0.6 | 0.43 | 0.77 | 109 | 196428 | Y |
| PFKL | missense | HbA1c | 1.93E-09 | 0.11 | 0.07 | 0.14 | 2798 | 196428 | Y |
| AXL | missense | HbA1c | 3.61E-09 | -0.09 | -0.12 | -0.06 | 3754 | 196428 | Y |
| LCAT | missense | HbA1c | 4.31E-09 | -0.31 | -0.42 | -0.21 | 299 | 196428 | Y |
| HNF1A | pLOF | HbA1c | 5.97E-09 | 0.91 | 0.6 | 1.21 | 35 | 196428 | Y |
| TMC8 | missense | HbA1c | 7.57E-09 | 0.16 | 0.1 | 0.21 | 1180 | 196428 | Y |
| APEH | missense | HbA1c | 2.05E-08 | 0.35 | 0.23 | 0.47 | 220 | 196428 | Y |
| PLA2G12A | missense | HbA1c | 3.4E-07 | -0.08 | -0.11 | -0.05 | 3616 | 196428 | Y |
| PFAS | missense | HbA1c | 6.06E-07 | -0.06 | -0.08 | -0.03 | 6819 | 196428 | Y |
| PTPRH | pLOF | HbA1c | 1.17E-06 | 0.11 | 0.07 | 0.16 | 1581 | 196428 | N |
| PLD1 | missense | HbA1c | 1.69E-06 | 0.08 | 0.05 | 0.12 | 2766 | 196428 | N |
| TNRC6B | pLOF | HbA1c | 1.91E-06 | 0.75 | 0.44 | 1.06 | 34 | 196428 | N |
| EPB41 | pLOF | HbA1c | 2.01E-06 | -0.4 | -0.57 | -0.24 | 118 | 196428 | N |
| APOB | pLOF | HbA1c | 3.89E-06 | 0.26 | 0.15 | 0.37 | 271 | 196428 | N |
| TNFRSF13B | missense | HbA1c | 7.24E-06 | -0.08 | -0.11 | -0.04 | 2944 | 196428 | N |
| SLC4A1 | missense | HbA1c | 8.8E-06 | -0.17 | -0.25 | -0.1 | 553 | 196428 | N |
| HK1 | missense | HbA1c | 4.35E-05 | -0.17 | -0.25 | -0.09 | 521 | 196428 | N |
| BLVRB | missense | HbA1c | 1.01E-04 | -0.07 | -0.1 | -0.03 | 2916 | 196428 | N |
| PLD1 | pLOF | HbA1c | 1.38E-04 | 0.23 | 0.11 | 0.35 | 230 | 196428 | N |
| GIGYF1 | pLOF | HbA1c | 2.10E-04 | 0.47 | 0.22 | 0.71 | 54 | 196428 | N |
| EPB42 | pLOF | HbA1c | 8.68E-04 | -0.28 | -0.44 | -0.12 | 121 | 196428 | N |
| CARHSP1 | missense | HbA1c | 0.003 | -0.1 | -0.17 | -0.04 | 715 | 196428 | N |
| TFR2 | missense | HbA1c | 0.004 | -0.1 | -0.16 | -0.03 | 782 | 196428 | N |
| PDX1 | missense | HbA1c | 0.005 | 0.04 | 0.01 | 0.07 | 3626 | 196428 | N |

## Supplementary Table 7: T2D associations tested in men and women separately

Results for variant sets identified in the primary analysis are shown, no additional significant associations were found.

| **gene** | **Variant set** | **title** | **pvalue** | **OR** | **95% CI-** | **95% CI+** | **n carrier** | **n carrier cases** | **n expected** | **N** | **significant in sex-stratified** |
| --- | --- | --- | --- | --- | --- | --- | --- | --- | --- | --- | --- |
| PAM | missense | T2D men | 7.41x10^-9^ | 1.33 | 1.21 | 1.47 | 4247 | 492 | 383.35 | 167178 | Y |
| GCK | missense | T2D men | 3.12x10^-4^ | 2.63 | 1.55 | 4.45 | 93 | 18 | 8.39 | 167178 | N |
| GIGYF1 | pLOF | T2D men | 3.72x10^-10^ | 5.01 | 3.03 | 8.30 | 76 | 23 | 6.86 | 167178 | Y |
| GCK | pLOF | T2D men | 3.96x10^-7^ | 11.23 | 4.41 | 28.61 | 19 | 10 | 1.71 | 167178 | Y |
| HNF1A | pLOF | T2D men | 1.41x10^-5^ | 4.84 | 2.38 | 9.87 | 37 | 12 | 3.34 | 167178 | N |
| TNRC6B | pLOF | T2D men | 0.001 | 3.56 | 1.64 | 7.71 | 36 | 9 | 3.25 | 167178 | N |
| GCK | missense | T2D women | 8.72x10^-6^ | 3.39 | 1.98 | 5.80 | 109 | 16 | 5.31 | 196428 | N |
| PAM | missense | T2D women | 7.15x10^-5^ | 1.27 | 1.13 | 1.43 | 5099 | 307 | 248.40 | 196428 | N |
| GCK | pLOF | T2D women | 4.39x10^-10^ | 17.45 | 7.11 | 42.85 | 21 | 9 | 1.02 | 196428 | Y |
| HNF1A | pLOF | T2D women | 1.47x10^-5^ | 5.91 | 2.65 | 13.21 | 36 | 8 | 1.75 | 196428 | N |
| TNRC6B | pLOF | T2D women | 1.78x10^-5^ | 5.83 | 2.61 | 13.04 | 35 | 8 | 1.71 | 196428 | N |
| GIGYF1 | pLOF | T2D women | 0.03 | 2.66 | 1.12 | 6.29 | 55 | 6 | 2.68 | 196428 | N |

## Supplementary Tables 8 – 10: Meta-analysis of sex-stratified results

Meta-analysis was performed on the sex-stratified results, and heterogeneity tested using Cochrane’s Q test. Effect sizes and p-values are shown for significant results along with a heterogeneity p-value (HetPVal). Supplied as excel files.

## Supplementary Table 8: Glucose meta-analysis in men and women

## Supplementary Table 9: HbA1c meta-analysis in men and women

## Supplementary Table 10: T2D meta-analysis in men and women

## Supplementary Table 11: Glucose and HbA1c associations of significant variant sets in other populations

Results for significant variant sets and variant sets identified in the primary analysis are shown for South Asian, African, and East Asian ancestry populations. Only variant sets with ≥ 10 carriers measured were tested and, within each population, a p-value threshold corrected for the number of variant sets tested was used.

| **South Asian** | | | | | | | | |
| --- | --- | --- | --- | --- | --- | --- | --- | --- |
| **gene** | **variant set** | **title** | **pvalue** | **effect (SD)** | **95% CI-** | **95% CI+** | **N carrier measured** | **N** |
| G6PC2 | missense | glucose | 8.31E-04 | -0.35 | -0.56 | -0.15 | 120 | 7553 |
| PFKM | missense | HbA1c | 0.001 | -0.36 | -0.57 | -0.14 | 87 | 7553 |
| TMC8 | missense | HbA1c | 0.010 | 0.27 | 0.07 | 0.48 | 100 | 7553 |
| SLC4A1 | missense | HbA1c | 0.10 | -0.2 | -0.44 | 0.04 | 74 | 7553 |
| PIEZO1 | missense | HbA1c | 0.14 | -0.08 | -0.18 | 0.03 | 408 | 7553 |
| G6PC2 | missense | HbA1c | 0.16 | -0.13 | -0.31 | 0.05 | 133 | 7553 |
| CARHSP1 | missense | HbA1c | 0.19 | 0.43 | -0.21 | 1.08 | 10 | 7553 |
| PLD1 | missense | HbA1c | 0.29 | -0.12 | -0.34 | 0.1 | 88 | 7553 |
| PFAS | missense | HbA1c | 0.45 | -0.07 | -0.26 | 0.11 | 121 | 7553 |
| PDX1 | missense | HbA1c | 0.45 | -0.07 | -0.26 | 0.11 | 120 | 7553 |
| AXL | missense | HbA1c | 0.58 | 0.06 | -0.15 | 0.27 | 94 | 7553 |
| BLVRB | missense | HbA1c | 0.62 | 0.14 | -0.41 | 0.68 | 14 | 7553 |
| TFR2 | missense | HbA1c | 0.73 | -0.11 | -0.72 | 0.5 | 11 | 7553 |
| ANK1 | missense | HbA1c | 0.73 | -0.03 | -0.19 | 0.13 | 161 | 7553 |
| HK1 | missense | HbA1c | 0.77 | -0.06 | -0.42 | 0.31 | 32 | 7553 |
| AMPD3 | missense | HbA1c | 0.86 | 0.02 | -0.17 | 0.21 | 117 | 7553 |
| APEH | missense | HbA1c | 0.88 | 0.04 | -0.47 | 0.55 | 16 | 7553 |
| PFKL | missense | HbA1c | 0.88 | 0.02 | -0.25 | 0.29 | 59 | 7553 |
| PLA2G12A | missense | HbA1c | 0.92 | -0.02 | -0.44 | 0.39 | 24 | 7553 |
| APOB | pLOF | HbA1c | 0.07 | 0.57 | -0.05 | 1.18 | 11 | 7553 |
| PTPRH | pLOF | HbA1c | 0.08 | 0.23 | -0.02 | 0.48 | 67 | 7553 |
| EPB42 | pLOF | HbA1c | 0.58 | 0.11 | -0.27 | 0.49 | 29 | 7553 |
| EPB41 | pLOF | HbA1c | 0.93 | 0.03 | -0.62 | 0.67 | 10 | 7553 |
| PLD1 | pLOF | HbA1c | 0.99 | 0 | -0.59 | 0.59 | 12 | 7553 |
| **African** | | | | | | | | |
| **gene** | **variant set** | **title** | **pvalue** | **effect (SD)** | **95% CI-** | **95% CI+** | **N carrier measured** | **N** |
| G6PC2 | missense | glucose | 0.01 | -0.34 | -0.61 | -0.08 | 66 | 6221 |
| SLC4A1 | missense | HbA1c | 0.10 | -0.24 | -0.54 | 0.05 | 60 | 6221 |
| APEH | missense | HbA1c | 0.12 | 0.51 | -0.14 | 1.16 | 12 | 6221 |
| TFR2 | missense | HbA1c | 0.16 | -0.45 | -1.08 | 0.18 | 13 | 6221 |
| HK1 | missense | HbA1c | 0.32 | 0.37 | -0.35 | 1.08 | 10 | 6221 |
| PLD1 | missense | HbA1c | 0.33 | 0.14 | -0.14 | 0.42 | 66 | 6221 |
| BLVRB | missense | HbA1c | 0.33 | 0.22 | -0.23 | 0.68 | 25 | 6221 |
| PFKM | missense | HbA1c | 0.37 | -0.2 | -0.63 | 0.23 | 27 | 6221 |
| AXL | missense | HbA1c | 0.43 | 0.16 | -0.24 | 0.57 | 31 | 6221 |
| PLA2G12A | missense | HbA1c | 0.45 | 0.26 | -0.42 | 0.94 | 11 | 6221 |
| AMPD3 | missense | HbA1c | 0.51 | 0.07 | -0.15 | 0.29 | 107 | 6221 |
| PFAS | missense | HbA1c | 0.61 | -0.05 | -0.22 | 0.13 | 166 | 6221 |
| ANK1 | missense | HbA1c | 0.68 | 0.06 | -0.24 | 0.37 | 55 | 6221 |
| TMC8 | missense | HbA1c | 0.70 | -0.07 | -0.41 | 0.27 | 44 | 6221 |
| G6PC2 | missense | HbA1c | 0.82 | -0.04 | -0.34 | 0.27 | 55 | 6221 |
| PIEZO1 | missense | HbA1c | 0.85 | -0.01 | -0.13 | 0.11 | 408 | 6221 |
| PDX1 | missense | HbA1c | 0.89 | -0.03 | -0.53 | 0.46 | 21 | 6221 |
| PFKL | missense | HbA1c | 0.94 | -0.01 | -0.36 | 0.33 | 43 | 6221 |
| PTPRH | pLOF | HbA1c | 0.19 | -0.34 | -0.84 | 0.17 | 20 | 6221 |
| **East Asian** | | | | | | | | |
| **gene** | **variant set** | **title** | **pvalue** | **effect (SD)** | **95% CI-** | **95% CI+** | **N carrier measured** | **N** |
| MYH6 | missense | HbA1c | 1.11x10^-6^ | -0.75 | -1.06 | -0.45 | 39 | 1315 |
| AMPD3 | missense | HbA1c | 0.003 | 0.49 | 0.16 | 0.81 | 33 | 1315 |
| ANK1 | missense | HbA1c | 0.29 | 0.24 | -0.2 | 0.68 | 18 | 1315 |
| TFR2 | missense | HbA1c | 0.36 | -0.27 | -0.86 | 0.31 | 10 | 1315 |
| PIEZO1 | missense | HbA1c | 0.41 | -0.08 | -0.27 | 0.11 | 99 | 1315 |
| PLD1 | missense | HbA1c | 0.88 | 0.02 | -0.29 | 0.33 | 37 | 1315 |
| PFKL | missense | HbA1c | 0.91 | 0.03 | -0.47 | 0.53 | 14 | 1315 |
| PFAS | missense | HbA1c | 0.94 | 0.01 | -0.34 | 0.37 | 28 | 1315 |

## Supplementary Table 12: T2D associations of significant variants in other populations

Results for variant sets significant in our primary analysis of T2D are shown are shown for South Asian, African, and East Asian ancestry populations. Only variant sets with ≥ 1 expected case carriers were tested.

| **South Asian** | | | | | | | | | |
| --- | --- | --- | --- | --- | --- | --- | --- | --- | --- |
| **gene** | **variant set** | **title** | **pvalue** | **OR** | **95% CI-** | **95% CI+** | **N** | **n carrier cases** | **n expected** |
| PAM | missense | T2D | 0.25 | 1.38 | -0.23 | 0.88 | 7553 | 20 | 15.86 |
| **African** | | | | | | | | | |
| **gene** | **variant set** | **title** | **pvalue** | **OR** | **95% CI-** | **95% CI+** | **N** | **n carrier cases** | **n expected** |
| PAM | missense | T2D | 0.09 | 1.68 | -0.09 | 1.13 | 6221 | 16 | 10.35 |
| **East Asian** | | | | | | | | | |
| too rare to test | | | | | | | | | |

## Supplementary Table 13: Conditional analysis for *GIGYF1* and *GCK* variants

| **gene** | **variant set** | **title** | **adjustment** | **pvalue** | **effect (beta/OR)** | **95% CI -** | **95% CI +** |
| --- | --- | --- | --- | --- | --- | --- | --- |
| GIGYF1 | pLOF | glucose | GCK pLOF and missense | 4.26x10^-12^ | 0.62 | 0.44 | 0.79 |
| GIGYF1 | pLOF | HbA1c | GCK pLOF and missense | 1.2x10^-14^ | 0.64 | 0.48 | 0.80 |
| GIGYF1 | pLOF | T2D | GCK pLOF and missense | 5.83x10^-11^ | 4.16 | 2.72 | 6.38 |

## Supplementary Table 14: Glucose, Hba1c and T2D associations for all significant variant sets

For all variant sets significantly associated with at least one diabetes-related trait in our primary analysis, associations with the other traits are shown.

| **Gene** | **Variant set** | **Pvalue glucose** | **Beta glucose** | **Pvalue hba1c** | **Beta hba1c** | **Pvalue T2D** | **OR T2D** |
| --- | --- | --- | --- | --- | --- | --- | --- |
| GCK | pLOF | 1.56E-09 | 0.999 | 2.64E-17 | 1.292 | 2.96E-15 | 14.16 |
| HNF1A | pLOF | 0.01 | 0.317 | 2.14E-07 | 0.592 | 1.23E-09 | 5.27 |
| GIGYF1 | pLOF | 4.42E-12 | 0.616 | 1.28E-14 | 0.639 | 6.14E-11 | 4.15 |
| GCK | missense CADD ≥ 25 | 6.15E-11 | 0.487 | 1.86E-17 | 0.565 | 1.7E-08 | 2.96 |
| PAM | missense CADD ≥ 25 | 0.92 | 0.001 | 0.009 | 0.026 | 2.26E-12 | 1.31 |
| IRS2 | pLOF | 0.07 | 0.320 | 1.58E-05 | 0.687 | 9.45E-06 | 5.88 |
| TNRC6B | pLOF | 4.01E-05 | 0.507 | 2.36E-07 | 0.582 | 2E-07 | 4.44 |
| PDX1 | missense CADD ≥ 25 | 0.02 | 0.029 | 2.54E-07 | 0.060 | 3.99E-05 | 1.21 |
| PFAS | missense CADD ≥ 25 | 0.32 | 0.009 | 2.09E-08 | -0.048 | 4.43E-04 | 0.88 |
| APOB | pLOF | 0.36 | 0.043 | 6.94E-08 | 0.232 | 0.02 | 1.42 |
| PLD1 | pLOF | 0.14 | 0.073 | 2.99E-07 | 0.231 | 0.01 | 1.50 |
| PTPRH | pLOF | 0.69 | 0.008 | 4.39E-10 | 0.109 | 0.10 | 1.12 |
| BLVRB | missense CADD ≥ 25 | 0.49 | 0.010 | 2.53E-08 | -0.071 | 0.30 | 0.95 |
| PLA2G12A | missense CADD ≥ 25 | 0.39 | 0.011 | 5.74E-11 | -0.076 | 0.09 | 0.92 |
| AMPD3 | missense CADD ≥ 25 | 0.82 | 0.003 | 7.28E-34 | 0.127 | 0.42 | 1.04 |
| CARHSP1 | missense CADD ≥ 25 | 0.86 | -0.005 | 6.78E-07 | -0.127 | 0.18 | 0.86 |
| LCAT | missense CADD ≥ 25 | 0.81 | -0.011 | 1.29E-11 | -0.268 | 0.31 | 0.83 |
| RHAG | pLOF | 0.10 | -0.126 | 3.31E-34 | -0.858 | 0.21 | 0.64 |
| ANK1 | missense CADD ≥ 25 | 0.46 | -0.012 | 3.12E-19 | -0.129 | 0.31 | 0.94 |
| HK1 | missense CADD ≥ 25 | 0.39 | 0.028 | 1.08E-07 | -0.159 | 0.14 | 1.19 |
| AXL | missense CADD ≥ 25 | 0.93 | 0.001 | 4.11E-12 | -0.080 | 0.28 | 0.95 |
| SLC4A1 | missense CADD ≥ 25 | 0.31 | 0.032 | 1.76E-07 | -0.154 | 0.35 | 1.12 |
| TFR2 | missense CADD ≥ 25 | 0.73 | 0.009 | 4.24E-07 | -0.124 | 0.45 | 0.92 |
| TMC8 | missense CADD ≥ 25 | 0.25 | -0.026 | 3.01E-08 | 0.114 | 0.41 | 0.93 |
| EPB42 | pLOF | 0.46 | -0.050 | 6.11E-07 | -0.307 | 0.42 | 1.21 |
| TNFRSF13B | missense CADD ≥ 25 | 0.98 | 0.000 | 4.63E-09 | -0.075 | 0.71 | 0.98 |
| PFKM | missense CADD ≥ 25 | 0.52 | 0.018 | 2.16E-28 | -0.283 | 0.27 | 0.88 |
| EPB41 | pLOF | 0.58 | 0.038 | 3.14E-17 | -0.529 | 0.26 | 1.30 |
| PFKL | missense CADD ≥ 25 | 0.82 | 0.003 | 2.69E-14 | 0.100 | 0.86 | 1.01 |
| APEH | missense CADD ≥ 25 | 0.30 | 0.052 | 1.86E-10 | 0.290 | 0.96 | 1.01 |
| G6PC2 | missense CADD ≥ 25 | 4.62E-83 | -0.266 | 6.71E-45 | -0.179 | 0.97 | 1.00 |
| PLD1 | missense CADD ≥ 25 | 0.54 | 0.009 | 1.51E-10 | 0.084 | 0.89 | 1.01 |
| PIEZO1 | missense CADD ≥ 25 | 0.05 | -0.013 | 9.9E-133 | -0.147 | 0.52 | 1.02 |

## Supplementary Table 15: OMIM disease associations for genes with a glucose, HbA1c or T2D association

Genes associated with glucose, HbA1c and/or T2D that are implicated in Mendelian disease according to OMIM [2].

| **gene** | **Variant set** | **OMIM ID** | **OMIM Phenotype** |
| --- | --- | --- | --- |
| AMPD3 | missense CADD ≥ 25 | 102772 | [AMP deaminase deficiency, erythrocytic] |
| ANK1 | missense CADD ≥ 25 | 612641 | Spherocytosis, type 1 |
| APOB | pLOF | 107730 | Hypobetalipoproteinemia : Hypercholesterolemia, familial, 2 |
| EPB41 | pLOF | 130500 | Elliptocytosis-1 |
| EPB42 | pLOF | 177070 | Spherocytosis, type 5 |
| GCK | pLOF | 138079 | MODY, type II : Hyperinsulinemic hypoglycemia, familial, 3 : Diabetes mellitus, permanent neonatal : Diabetes mellitus, noninsulin-dependent, late onset |
| GCK | missense CADD ≥ 25 | 138079 | MODY, type II : Hyperinsulinemic hypoglycemia, familial, 3 : Diabetes mellitus, permanent neonatal : Diabetes mellitus, noninsulin-dependent, late onset |
| HK1 | missense CADD ≥ 25 | 142600 | Hemolytic anemia due to hexokinase deficiency : Retinitis pigmentosa 79 : Neuropathy, hereditary motor and sensory, Russe type |
| HNF1A | pLOF | 142410 | Renal cell carcinoma : MODY, type III : Hepatic adenoma, somatic : Diabetes mellitus, insulin-dependent, 20 |
| LCAT | missense CADD ≥ 25 | 606967 | Fish-eye disease : Norum disease |
| PDX1 | missense CADD ≥ 25 | 600733 | Lacticacidemia due to PDX1 deficiency : MODY, type IV : Pancreatic agenesis 1 |
| PFKL | missense CADD ≥ 25 | 171860 | Hemolytic anemia due to phosphofructokinase deficiency |
| PFKM | missense CADD ≥ 25 | 610681 | Glycogen storage disease VII |
| PIEZO1 | missense CADD ≥ 25 | 611184 | Lymphatic malformation 6 : Dehydrated hereditary stomatocytosis with or without pseudohyperkalemia and/or perinatal edema |
| PLD1 | missense CADD ≥ 25 | 602382 | Cardiac valvular defect, developmental |
| PLD1 | pLOF | 602382 | Cardiac valvular defect, developmental |
| RHAG | pLOF | 180297 | Overhydrated hereditary stomatocytosis : Anemia, hemolytic, Rh-null, regulator type |
| SLC4A1 | missense CADD ≥ 25 | 109270 | Spherocytosis, type 4 : [Blood group, Wright] : Renal tubular acidosis, distal, AR : [Blood group, Waldner] : Renal tubular acidosis, distal, AD : [Blood group, Swann] : Ovalocytosis, SA type : [Blood group, Froese] : Cryohydrocytosis : [Blood group, Diego] : [Malaria, resistance to] |
| TFR2 | missense CADD ≥ 25 | 604720 | Hemochromatosis, type 3 |
| TMC8 | missense CADD ≥ 25 | 605829 | Epidermodysplasia verruciformis 2 |
| TNFRSF13B | missense CADD ≥ 25 | 604907 | Immunoglobulin A deficiency 2 : Immunodeficiency, common variable, 2 |

## Supplementary Table 16: HbA1c-associated genes and red blood cell traits (supplied as an excel file).

## Supplementary Table 17: Glucose and HbA1c associations adjusted for BMI

| **gene** | **Variant set** | **title** | **adjustment** | **pvalue** | **Effect (SD)** | **95% CI -** | **95% CI +** | **N carrier measured** |
| --- | --- | --- | --- | --- | --- | --- | --- | --- |
| G6PC2 | missense CADD ≥ 25 | glucose | unadjusted | 4.62E-83 | -0.27 | -0.29 | -0.24 | 5128 |
| G6PC2 | missense CADD ≥ 25 | glucose | BMI | 6.63E-84 | -0.26 | -0.29 | -0.24 | 5110 |
| GCK | missense CADD ≥ 25 | glucose | unadjusted | 6.15E-11 | 0.49 | 0.34 | 0.63 | 173 |
| GCK | missense CADD ≥ 25 | glucose | BMI | 3.32E-11 | 0.49 | 0.34 | 0.63 | 173 |
| GCK | pLOF | glucose | unadjusted | 1.56E-09 | 1 | 0.67 | 1.32 | 35 |
| GCK | pLOF | glucose | BMI | 3.82E-09 | 0.96 | 0.64 | 1.28 | 35 |
| GIGYF1 | pLOF | glucose | unadjusted | 4.42E-12 | 0.62 | 0.44 | 0.79 | 121 |
| GIGYF1 | pLOF | glucose | BMI | 1.02E-10 | 0.57 | 0.4 | 0.74 | 120 |
| G6PC2 | missense CADD ≥ 25 | HbA1c | unadjusted | 6.71E-45 | -0.18 | -0.2 | -0.15 | 5574 |
| G6PC2 | missense CADD ≥ 25 | HbA1c | BMI | 3.42E-47 | -0.18 | -0.2 | -0.15 | 5555 |
| GCK | missense CADD ≥ 25 | HbA1c | unadjusted | 1.86E-17 | 0.56 | 0.43 | 0.69 | 201 |
| GCK | missense CADD ≥ 25 | HbA1c | BMI | 9.48E-18 | 0.55 | 0.43 | 0.68 | 201 |
| GCK | pLOF | HbA1c | unadjusted | 2.64E-17 | 1.29 | 0.99 | 1.59 | 38 |
| GCK | pLOF | HbA1c | BMI | 5.94E-17 | 1.24 | 0.95 | 1.53 | 38 |
| GIGYF1 | pLOF | HbA1c | unadjusted | 1.28E-14 | 0.64 | 0.48 | 0.8 | 129 |
| GIGYF1 | pLOF | HbA1c | BMI | 3.59E-13 | 0.59 | 0.43 | 0.75 | 128 |
| HNF1A | pLOF | HbA1c | unadjusted | 2.14E-07 | 0.59 | 0.37 | 0.82 | 68 |
| HNF1A | pLOF | HbA1c | BMI | 4.60E-08 | 0.61 | 0.39 | 0.82 | 68 |
| PDX1 | missense CADD ≥ 25 | HbA1c | unadjusted | 2.54E-07 | 0.06 | 0.04 | 0.08 | 6694 |
| PDX1 | missense CADD ≥ 25 | HbA1c | BMI | 2.88E-08 | 0.06 | 0.04 | 0.08 | 6673 |
| PFAS | missense CADD ≥ 25 | HbA1c | unadjusted | 2.09E-08 | -0.05 | -0.06 | -0.03 | 12621 |
| PFAS | missense CADD ≥ 25 | HbA1c | BMI | 5.11E-09 | -0.05 | -0.06 | -0.03 | 12573 |
| TNRC6B | pLOF | HbA1c | unadjusted | 2.36E-07 | 0.58 | 0.36 | 0.8 | 70 |
| TNRC6B | pLOF | HbA1c | BMI | 5.16E-07 | 0.55 | 0.33 | 0.76 | 70 |

## Supplementary Table 18: T2D associations adjusted for BMI

| **gene** | **variant set** | **title** | **adjustment** | **pvalue** | **OR** | **95% CI -** | **95% CI +** | **n cases** |
| --- | --- | --- | --- | --- | --- | --- | --- | --- |
| GCK | missense CADD ≥ 25 | T2D | unadjusted | 1.7E-08 | 2.96 | 2.03 | 4.32 | 24695 |
| GCK | missense CADD ≥ 25 | T2D | BMI | 2.07E-09 | 3.34 | 2.25 | 4.95 | 24504 |
| GCK | pLOF | T2D | unadjusted | 2.96E-15 | 14.16 | 7.33 | 27.34 | 24695 |
| GCK | pLOF | T2D | BMI | 4.17E-15 | 16.27 | 8.11 | 32.64 | 24504 |
| GIGYF1 | pLOF | T2D | unadjusted | 6.14E-11 | 4.15 | 2.71 | 6.37 | 24695 |
| GIGYF1 | pLOF | T2D | BMI | 1.38E-09 | 4.08 | 2.59 | 6.43 | 24504 |
| HNF1A | pLOF | T2D | unadjusted | 1.23E-09 | 5.27 | 3.08 | 9 | 24695 |
| HNF1A | pLOF | T2D | BMI | 2.94E-11 | 6.89 | 3.9 | 12.16 | 24504 |
| PAM | missense CADD ≥ 25 | T2D | unadjusted | 2.26E-12 | 1.31 | 1.21 | 1.41 | 24695 |
| PAM | missense CADD ≥ 25 | T2D | BMI | 4.62E-14 | 1.35 | 1.25 | 1.46 | 24504 |
| TNRC6B | pLOF | T2D | unadjusted | 2.00E-07 | 4.44 | 2.53 | 7.79 | 24695 |
| TNRC6B | pLOF | T2D | BMI | 1.89E-07 | 4.84 | 2.67 | 8.75 | 24504 |

## Supplementary Table 19: SAIGE-gene results for variant sets of interest

SAIGE-Gene was run in the European ancestry population including related individuals. Whether the association is significant at the thresholds used in our primary analysis (p ≤ 7.82 x 10^-7^ for biomarkers and p ≤ 1.46 x 10^-6^ for T2D) is indicated.

| **Gene** | **Variant set** | **title** | **pvalue** | **significant** |
| --- | --- | --- | --- | --- |
| G6PC2 | missense CADD ≥ 25 | glucose | 1.30E-99 | Y |
| GCK | missense CADD ≥ 25 | glucose | 2.01E-11 | Y |
| GCK | pLOF | glucose | 6.56E-11 | Y |
| GIGYF1 | pLOF | glucose | 8.32E-13 | Y |
| G6PC2 | missense CADD ≥ 25 | HbA1c | 1.17E-62 | Y |
| GCK | missense CADD ≥ 25 | HbA1c | 3.20E-25 | Y |
| GCK | pLOF | HbA1c | 1.75E-20 | Y |
| GIGYF1 | pLOF | HbA1c | 1.92E-22 | Y |
| HNF1A | pLOF | HbA1c | 1.52E-08 | Y |
| PDX1 | missense CADD ≥ 25 | HbA1c | 9.05E-12 | Y |
| PFAS | missense CADD ≥ 25 | HbA1c | 2.00E-10 | Y |
| TNRC6B | pLOF | HbA1c | 6.85E-06 | N |
| GCK | missense CADD ≥ 25 | T2D | 2.33E-09 | Y |
| GCK | pLOF | T2D | 3.27E-15 | Y |
| GIGYF1 | pLOF | T2D | 7.34E-09 | Y |
| HNF1A | pLOF | T2D | 3.29E-09 | Y |
| PAM | missense CADD ≥ 25 | T2D | 5.60E-14 | Y |
| TNRC6B | pLOF | T2D | 4.77E-05 | N |

## Supplementary Table 20: Glucose and HbA1c associations excluding diagnosed diabetics

Burden tests for glucose and HbA1c were performed for variants sets of interest excluding individuals diagnosed with any form of diabetes (ICD10 codes E10, E11, E12, E13 and E14).

| **gene** | **variant set** | **title** | **pvalue** | **Beta (SD)** | **95% CI -** | **95% CI +** | **n carrier measured** |
| --- | --- | --- | --- | --- | --- | --- | --- |
| G6PC2 | missense | glucose | 4.62E-83 | -0.27 | -0.29 | -0.24 | 5128 |
| G6PC2 | missense | glucose non-diabetic | 9.03E-97 | -0.28 | -0.31 | -0.25 | 4758 |
| GCK | missense | glucose | 6.15E-11 | 0.49 | 0.34 | 0.63 | 173 |
| GCK | missense | glucose non-diabetic | 2.42E-07 | 0.40 | 0.25 | 0.55 | 143 |
| GCK | pLOF | glucose | 1.56E-09 | 1.00 | 0.67 | 1.32 | 35 |
| GCK | pLOF | glucose non-diabetic | 0.02 | 0.47 | 0.07 | 0.87 | 20 |
| GIGYF1 | pLOF | glucose | 4.42E-12 | 0.62 | 0.44 | 0.79 | 121 |
| GIGYF1 | pLOF | glucose non-diabetic | 2.95E-08 | 0.53 | 0.34 | 0.72 | 92 |
| G6PC2 | missense | HbA1c | 6.71E-45 | -0.18 | -0.20 | -0.15 | 5574 |
| G6PC2 | missense | HbA1c non-diabetic | 1.45E-55 | -0.19 | -0.21 | -0.17 | 5171 |
| GCK | missense | HbA1c | 1.86E-17 | 0.56 | 0.43 | 0.69 | 201 |
| GCK | missense | HbA1c non-diabetic | 5.46E-11 | 0.44 | 0.31 | 0.57 | 166 |
| GCK | pLOF | HbA1c | 2.64E-17 | 1.29 | 0.99 | 1.59 | 38 |
| GCK | pLOF | HbA1c non-diabetic | 5.95E-05 | 0.79 | 0.41 | 1.18 | 19 |
| GIGYF1 | pLOF | HbA1c | 1.28E-14 | 0.64 | 0.48 | 0.80 | 129 |
| GIGYF1 | pLOF | HbA1c non-diabetic | 8.29E-07 | 0.43 | 0.26 | 0.60 | 99 |
| HNF1A | pLOF | HbA1c | 2.14E-07 | 0.59 | 0.37 | 0.82 | 68 |
| HNF1A | pLOF | HbA1c non-diabetic | 0.013 | 0.30 | 0.06 | 0.54 | 50 |
| PDX1 | missense | HbA1c | 2.54E-07 | 0.06 | 0.04 | 0.08 | 6694 |
| PDX1 | missense | HbA1c non-diabetic | 4.54E-04 | 0.04 | 0.02 | 0.06 | 6132 |
| PFAS | missense | HbA1c | 2.09E-08 | -0.05 | -0.06 | -0.03 | 12621 |
| PFAS | missense | HbA1c non-diabetic | 1.9E-07 | -0.04 | -0.06 | -0.03 | 11790 |
| TNRC6B | pLOF | HbA1c | 2.36E-07 | 0.58 | 0.36 | 0.80 | 70 |
| TNRC6B | pLOF | HbA1c non-diabetic | 0.009 | 0.31 | 0.08 | 0.55 | 52 |

## Supplementary Table 21: Associations for pathogenic variants in MODY genes with glucose and HbA1c

Variants annotated as pathogenic or likely pathogenic in ClinVar were tested alone or combined with variants significant in the primary analysis.

| **gene** | **variant set** | **title** | **pvalue** | **effect (SD)** | **95% CI-** | **95% CI+** | **n carrier measured** |
| --- | --- | --- | --- | --- | --- | --- | --- |
| GCK | missense | HbA1c | 1.93E-17 | 0.56 | 0.43 | 0.69 | 201 |
| GCK | missense | glucose | 6.95E-11 | 0.49 | 0.34 | 0.63 | 173 |
| GCK | missense+clinvar | HbA1c | 2.25E-30 | 0.70 | 0.58 | 0.82 | 237 |
| GCK | missense+clinvar | glucose | 3.2E-21 | 0.65 | 0.51 | 0.78 | 204 |
| GCK | clinvar | HbA1c | 1.86E-25 | 1.48 | 1.20 | 1.76 | 44 |
| GCK | clinvar | glucose | 4.01E-22 | 1.56 | 1.24 | 1.87 | 37 |
| GCK | pLOF+clinvar | HbA1c | 2.98E-34 | 1.34 | 1.13 | 1.56 | 73 |
| GCK | pLOF+clinvar | glucose | 3.27E-24 | 1.23 | 1.00 | 1.47 | 65 |
| GCK | pLOF | HbA1c | 2.64E-17 | 1.29 | 0.99 | 1.59 | 38 |
| GCK | pLOF | glucose | 1.56E-09 | 1.00 | 0.67 | 1.32 | 35 |
| HNF1A | pLOF | HbA1c | 2.14E-07 | 0.59 | 0.37 | 0.82 | 68 |
| HNF1A | pLOF | glucose | 0.01 | 0.32 | 0.08 | 0.56 | 64 |
| HNF1A | pLOF+clinvar | HbA1c | 3.7E-08 | 0.32 | 0.20 | 0.43 | 266 |
| HNF1A | pLOF+clinvar | glucose | 7.83E-04 | 0.21 | 0.09 | 0.34 | 240 |
| HNF1A | clinvar | HbA1c | 1.46E-04 | 0.25 | 0.12 | 0.38 | 204 |
| HNF1A | clinvar | glucose | 0.03 | 0.16 | 0.02 | 0.30 | 181 |
| PDX1 | missense | HbA1c | 2.72E-07 | 0.06 | 0.04 | 0.08 | 6694 |
| PDX1 | missense | glucose | 0.02 | 0.03 | 0.00 | 0.05 | 6079 |
| PDX1 | missense+clinvar | HbA1c | 2.43E-07 | 0.06 | 0.04 | 0.08 | 6695 |
| PDX1 | missense+clinvar | glucose | 0.02 | 0.03 | 0.00 | 0.05 | 6081 |
| PDX1 | clinvar | glucose | 0.12 | 0.11 | -0.03 | 0.25 | 190 |
| PDX1 | clinvar | HbA1c | 0.89 | -0.01 | -0.14 | 0.12 | 212 |

## Supplementary Table 22: Associations for pathogenic variants in MODY genes with T2D

Variants annotated as pathogenic or likely pathogenic in ClinVar were tested alone or combined with variants significant in the primary analysis.

| **gene** | **Variant set** | **title** | **pvalue** | **OR** | **95% CI-** | **95% CI+** | **n carrier cases** | **n expected** |
| --- | --- | --- | --- | --- | --- | --- | --- | --- |
| GCK | missense | T2D | 2.56E-08 | 2.90 | 2.00 | 4.23 | 34 | 13.71 |
| GCK | missense+clinvar | T2D | 6.83E-15 | 3.65 | 2.64 | 5.06 | 47 | 16.15 |
| GCK | clinvar | T2D | 3.12E-13 | 10.68 | 5.65 | 20.20 | 16 | 2.99 |
| GCK | pLOF | T2D | 2.96E-15 | 14.16 | 7.33 | 27.34 | 19 | 2.71 |
| GCK | pLOF+clinvar | T2D | 6.33E-23 | 11.22 | 6.94 | 18.14 | 30 | 5.09 |
| HNF1A | pLOF | T2D | 1.23E-09 | 5.27 | 3.08 | 9.00 | 20 | 4.95 |
| HNF1A | pLOF+clinvar | T2D | 1.76E-15 | 3.39 | 2.51 | 4.58 | 55 | 19.07 |
| HNF1A | clinvar | T2D | 4.86E-10 | 3.07 | 2.16 | 4.37 | 39 | 14.59 |
| PDX1 | missense | T2D | 4.95E-05 | 1.20 | 1.10 | 1.31 | 553 | 473.71 |
| PDX1 | missense+clinvar | T2D | 5.03E-05 | 1.20 | 1.10 | 1.31 | 553 | 473.85 |
| PDX1 | clinvar | T2D | 0.998 | 1.00 | 0.59 | 1.70 | 15 | 15.13 |

## Supplementary Table 23: Glucose and HbA1c associations from primary care data

Associations with glucose and HbA1c measurements obtained from primary care (GP) data. Whether the association is significant adjusting for the number of variant sets and traits tested (p ≤ 0.004) is indicated.

| **gene** | **Variant set** | **title** | **pvalue** | **beta** | **95% CI -** | **95% CI +** | **N carrier measured** | **significant** |
| --- | --- | --- | --- | --- | --- | --- | --- | --- |
| GIGYF1 | pLOF | Glucose GP | 2.1E-06 | 0.65 | 0.38 | 0.92 | 50 | Y |
| GCK | pLOF | Glucose GP | 1.41E-08 | 1.29 | 0.85 | 1.74 | 18 | Y |
| GCK | missense | Glucose GP | 0.00253 | 0.36 | 0.13 | 0.59 | 67 | Y |
| G6PC2 | missense | Glucose GP | 1.6E-20 | -0.19 | -0.23 | -0.15 | 2192 | Y |
| G6PC2 | missense | HbA1c GP | 4.14E-06 | -0.13 | -0.19 | -0.08 | 1169 | Y |
| GCK | pLOF | HbA1c GP | 3.9E-06 | 1.12 | 0.65 | 1.60 | 16 | Y |
| GCK | missense | HbA1c GP | 0.0625 | 0.26 | -0.01 | 0.53 | 50 | N |
| GIGYF1 | pLOF | HbA1c GP | 1.19E-05 | 0.74 | 0.41 | 1.07 | 33 | Y |
| HNF1A | pLOF | HbA1c GP | 5.68E-04 | 0.65 | 0.28 | 1.01 | 27 | Y |
| PDX1 | missense | HbA1c GP | 0.003 | 0.08 | 0.03 | 0.13 | 1385 | Y |
| PFAS | missense | HbA1c GP | 0.001 | -0.06 | -0.10 | -0.02 | 2679 | Y |
| TNRC6B | pLOF | HbA1c GP | 0.01 | 0.58 | 0.13 | 1.03 | 18 | N |

## Supplementary Table 24: T2D associations for novel genes in Geisinger Health System

For novel genes associated with diabetes traits in our primary analysis we tested relevant variant sets for association with T2D diagnosis in Geisinger Health System (GHS). Variant set definitions and analysis methods varied somewhat from our primary analysis (see Methods).

| **Phenotype** | **Gene** | **Variant set** | **pvalue** | **OR (95% CI)** | **MAF (%)** | **Genotype counts cases, RR\|RA\|AA genotypes** | **Genotype counts controls, RR\|RA\|AA genotypes** |
| --- | --- | --- | --- | --- | --- | --- | --- |
| T2D | GIGYF1 | pLOF; MAF < 1% | 0.01 | 1.8 (1.1, 2.8) | 0.05% | 25,808\|38\|0 | 63,697\|52\|0 |
| T2D | TNRC6B | pLOF; MAF < 1% | 0.66 | 1.1  (0.8, 1.4) | 0.16% | 25,775\|71\|0 | 63,531\|218\|0 |
| T2D | PFAS | pLOF plus deleterious missense; MAF < 1% | 0.29 | 1 (0.96, 1.1) | 1.78% | 24,898\|946\|2 | 61,512\|2,233\|4 |

## Supplementary Table 25: Association results when singletons are excluded

| **gene** | **Variant set** | **Included variants** | **phenotype** | **pvalue** | **effect (beta/OR)** | **n carrier** | **significant** |
| --- | --- | --- | --- | --- | --- | --- | --- |
| GCK | missense CADD ≥ 25 | All | T2D | 1.70E-08 | 2.96 | 202 | Y |
| GCK | missense CADD ≥ 25 | No singletons | T2D | 3.04E-04 | 2.29 | 166 | N |
| GCK | pLOF | All | T2D | 2.96E-15 | 14.16 | 40 | Y |
| GCK | pLOF | No singletons | T2D | 2.57E-07 | 10.88 | 21 | Y |
| GIGYF1 | pLOF | All | T2D | 6.14E-11 | 4.15 | 131 | Y |
| GIGYF1 | pLOF | No singletons | T2D | 2.91E-05 | 3.64 | 72 | N |
| HNF1A | pLOF | All | T2D | 1.23E-09 | 5.27 | 73 | Y |
| HNF1A | pLOF | No singletons | T2D | 2.34E-08 | 5.21 | 63 | Y |
| PAM | missense CADD ≥ 25 | All | T2D | 2.26E-12 | 1.31 | 9357 | Y |
| PAM | missense CADD ≥ 25 | No singletons | T2D | 4.1E-12 | 1.30 | 9257 | Y |
| TNRC6B | pLOF | All | T2D | 2E-07 | 4.44 | 71 | Y |
| TNRC6B | pLOF | No singletons | T2D | 0.69 | 1.36 | 23 | N |
| G6PC2 | missense CADD ≥ 25 | All | glucose | 4.62E-83 | -0.27 | 5128 | Y |
| G6PC2 | missense CADD ≥ 25 | No singletons | glucose | 1.12E-82 | -0.27 | 5103 | Y |
| GCK | missense CADD ≥ 25 | All | glucose | 6.15E-11 | 0.49 | 173 | Y |
| GCK | missense CADD ≥ 25 | No singletons | glucose | 6.98E-05 | 0.33 | 142 | N |
| GCK | pLOF | All | glucose | 1.56E-09 | 1.00 | 35 | Y |
| GCK | pLOF | No singletons | glucose | 0.0015 | 0.69 | 20 | N |
| GIGYF1 | pLOF | All | glucose | 4.42E-12 | 0.62 | 121 | Y |
| GIGYF1 | pLOF | No singletons | glucose | 1.54E-05 | 0.52 | 66 | N |
| G6PC2 | missense CADD ≥ 25 | All | HbA1c | 6.71E-45 | -0.18 | 5574 | Y |
| G6PC2 | missense CADD ≥ 25 | No singletons | HbA1c | 9.98E-45 | -0.18 | 5548 | Y |
| GCK | missense CADD ≥ 25 | All | HbA1c | 1.86E-17 | 0.56 | 201 | Y |
| GCK | missense CADD ≥ 25 | No singletons | HbA1c | 6.13E-09 | 0.43 | 165 | Y |
| GCK | pLOF | All | HbA1c | 2.64E-17 | 1.29 | 38 | Y |
| GCK | pLOF | No singletons | HbA1c | 1.16E-05 | 0.90 | 21 | N |
| GIGYF1 | pLOF | All | HbA1c | 1.28E-14 | 0.64 | 129 | Y |
| GIGYF1 | pLOF | No singletons | HbA1c | 5.47E-09 | 0.65 | 72 | Y |
| HNF1A | pLOF | All | HbA1c | 2.14E-07 | 0.59 | 68 | Y |
| HNF1A | pLOF | No singletons | HbA1c | 4.75E-06 | 0.56 | 59 | N |
| PDX1 | missense CADD ≥ 25 | All | HbA1c | 2.54E-07 | 0.06 | 6694 | Y |
| PDX1 | missense CADD ≥ 25 | No singletons | HbA1c | 4.75E-07 | 0.06 | 6662 | Y |
| PFAS | missense CADD ≥ 25 | All | HbA1c | 2.09E-08 | -0.05 | 12621 | Y |
| PFAS | missense CADD ≥ 25 | No singletons | HbA1c | 7.07E-08 | -0.05 | 12493 | Y |
| TNRC6B | pLOF | All | HbA1c | 2.36E-07 | 0.58 | 70 | Y |
| TNRC6B | pLOF | No singletons | HbA1c | 0.09 | 0.34 | 23 | N |

## Supplementary Table 26: Associations for variants in *SLC30A8* and *MC4R*

We examined glucose, HbA1c and T2D associations for *SLC30A8* and *MC4R*, genes reported to associate with T2D by Flannick and colleagues [3].

| **variant set** | **title** | **pvalue** | **effect (SD/OR)** | **95% CI -** | **95% CI +** | **n carrier measured** | **n carrier cases** | **n expected** |
| --- | --- | --- | --- | --- | --- | --- | --- | --- |
| SLC30A8 pLOF | HbA1c | 1.44E-05 | -0.28 | -0.41 | -0.15 | 213 |  |  |
| SLC30A8 pLOF | glucose | 0.003 | -0.20 | -0.33 | -0.07 | 209 |  |  |
| SLC30A8 pLOF | T2D | 0.03 | 0.42 | 0.20 | 0.91 |  | 7 | 15.81 |
| SLC30A8 missense | HbA1c | 6.16E-06 | -0.18 | -0.26 | -0.10 | 554 |  |  |
| SLC30A8 missense | glucose | 2.51E-04 | -0.16 | -0.25 | -0.08 | 493 |  |  |
| SLC30A8 missense | T2D | 0.06 | 0.69 | 0.47 | 1.01 | 577 | 28 | 39.15 |
| SLC30A8 pLOF+missense | HbA1c | 8.64E-10 | -0.21 | -0.28 | -0.14 | 767 |  |  |
| SLC30A8 pLOF+missense | glucose | 2.71E-06 | -0.17 | -0.25 | -0.10 | 702 |  |  |
| SLC30A8 pLOF+missense | T2D | 0.005 | 0.61 | 0.44 | 0.86 |  | 35 | 54.96 |
| MC4R missense | HbA1c | 0.19 | 0.04 | -0.02 | 0.10 | 872 |  |  |
| MC4R missense | glucose | 0.78 | 0.01 | -0.06 | 0.08 | 787 |  |  |
| MC4R missense | T2D | 0.27 | 1.15 | 0.90 | 1.46 |  | 73 | 62.56 |

## Supplementary Table 27: Drug target gene associations with HbA1c and T2D

HbA1c and T2D associations for pLOF and damaging missense variants in the genes encoding T2D drug targets.

**p ≤ 0.003 (significance correcting for 15 variant sets tested)

*p ≤ 0.05 (nominal significance)

| **Variant set** | **title** | **pvalue** | **Effect (beta/OR)** | **95% CI -** | **95% CI +** | **N carrier measured or N carrier cases/N expected** | **Significance** |
| --- | --- | --- | --- | --- | --- | --- | --- |
| DPP4 pLOF | HbA1c | 1.4E-05 | -0.20 | -0.29 | -0.11 | 424 | ** |
| GLP1R missense | HbA1c | 0.002 | 0.10 | 0.04 | 0.16 | 869 | ** |
| IGF1R pLOF | HbA1c | 0.01 | 0.25 | 0.06 | 0.44 | 91 | * |
| IGF1R missense | HbA1c | 0.02 | 0.05 | 0.01 | 0.09 | 2145 | * |
| DPP4 missense | HbA1c | 0.09 | -0.04 | -0.08 | 0.01 | 2085 |  |
| ABCC8 pLOF | HbA1c | 0.14 | 0.08 | -0.03 | 0.20 | 274 |  |
| KCNJ11 missense | HbA1c | 0.47 | 0.03 | -0.05 | 0.10 | 584 |  |
| SLC5A2 pLOF | HbA1c | 0.57 | -0.03 | -0.14 | 0.08 | 306 |  |
| INSR missense | HbA1c | 0.60 | 0.01 | -0.04 | 0.07 | 1253 |  |
| ABCC8 missense | HbA1c | 0.60 | 0.01 | -0.03 | 0.06 | 1718 |  |
| INSR pLOF | HbA1c | 0.64 | 0.04 | -0.12 | 0.20 | 133 |  |
| SLC5A2 missense | HbA1c | 0.73 | -0.01 | -0.06 | 0.04 | 1485 |  |
| PPARG pLOF | HbA1c | 0.80 | -0.06 | -0.52 | 0.40 | 16 |  |
| GLP1R pLOF | HbA1c | 0.83 | -0.02 | -0.22 | 0.18 | 86 |  |
| PPARG missense | HbA1c | 0.90 | 0.01 | -0.16 | 0.18 | 119 |  |
| KCNJ11 missense | T2D | 2.19E-04 | 1.65 | 1.26 | 2.15 | 63/42.27 | ** |
| PPARG pLOF | T2D | 0.007 | 5.08 | 1.56 | 16.54 | 4/1.09 | * |
| IGF1R missense | T2D | 0.01 | 1.22 | 1.05 | 1.43 | 181/152.18 | * |
| GLP1R missense | T2D | 0.02 | 1.32 | 1.04 | 1.67 | 78/61.94 | * |
| INSR pLOF | T2D | 0.03 | 1.80 | 1.06 | 3.06 | 16/9.16 | * |
| ABCC8 missense | T2D | 0.04 | 1.20 | 1.01 | 1.43 | 143/121.92 | * |
| PPARG missense | T2D | 0.06 | 1.77 | 0.98 | 3.17 | 13/8.41 |  |
| INSR missense | T2D | 0.16 | 0.84 | 0.67 | 1.07 | 77/89.08 |  |
| DPP4 missense | T2D | 0.36 | 1.08 | 0.92 | 1.27 | 159/148.32 |  |
| SLC5A2 pLOF | T2D | 0.39 | 0.81 | 0.50 | 1.31 | 18/21.98 |  |
| GLP1R pLOF | T2D | 0.58 | 1.24 | 0.57 | 2.72 | 7/5.97 |  |
| IGF1R pLOF | T2D | 0.68 | 1.17 | 0.56 | 2.42 | 8/6.65 |  |
| DPP4 pLOF | T2D | 0.74 | 0.94 | 0.65 | 1.37 | 30/30.53 |  |
| ABCC8 pLOF | T2D | 0.78 | 0.94 | 0.58 | 1.50 | 19/19.54 |  |
| SLC5A2 missense | T2D | 0.97 | 1.00 | 0.82 | 1.23 | 104/105.37 |  |

## Supplementary Table 28: *GIGYF1* pLOF associations with cholesterol levels

The association of *GIGYF1* pLOF with total and LDL cholesterol was tested 1) adjusting for use of cholesterol-lowering medication and 2) excluding those on cholesterol-lowering medication from the analysis.

| **gene** | **variant set** | **title** | **adjustment/subset** | **pvalue** | **beta** | **95% CI -** | **95% CI +** | **n carrier measured** |
| --- | --- | --- | --- | --- | --- | --- | --- | --- |
| GIGYF1 | pLOF | total cholesterol | unadjusted | 2.44E-12 | -0.61 | -0.78 | -0.44 | 128 |
| GIGYF1 | pLOF | LDL cholesterol | unadjusted | 2.40E-10 | -0.56 | -0.73 | -0.38 | 128 |
| GIGYF1 | pLOF | total cholesterol | adjusted cholesterol medication | 4.14E-11 | -0.52 | -0.67 | -0.36 | 128 |
| GIGYF1 | pLOF | LDL cholesterol | adjusted cholesterol medication | 4.68E-09 | -0.46 | -0.62 | -0.31 | 128 |
| GIGYF1 | pLOF | total cholesterol | exclude cholesterol medication | 3.70E-07 | -0.46 | -0.63 | -0.28 | 96 |
| GIGYF1 | pLOF | LDL cholesterol | exclude cholesterol medication | 1.54E-05 | -0.39 | -0.57 | -0.21 | 96 |

## Supplementary Table 29: rs221783 is an eQTL for *GIGYF1*

GTEx eQTL data for rs221783 in selected tissues. The nominal p-value is shown along with the slope from the linear regression (giving direction of effect) and standard error (SE) for the T allele. In all cases, rs221783 is highly or completely correlated with the best eQTL for *GIGYF1* as shown in the “R^2^ with best” column.

| **Tissue** | **rsid** | **GTEx variant id** | **gene name** | **pvalue** | **Slope (T allele)** | **Slope SE** | **rsid for best eQTL** | **R^2^ with best** |
| --- | --- | --- | --- | --- | --- | --- | --- | --- |
| Adipose - subcutaneous | rs221783 | chr7_100695291_T_C_b38 | *GIGYF1* | 1.57x10^-30^ | 0.35 | 0.03 | rs221781 | 1.00 |
| Lung | rs221783 | chr7_100695291_T_C_b38 | *GIGYF1* | 1.00x10^-17^ | 0.32 | 0.04 | rs221774 | 1.00 |
| Pancreas | rs221783 | chr7_100695291_T_C_b38 | *GIGYF1* | 6.40x10^-10^ | 0.38 | 0.06 | rs221786 | 0.96 |
| Thyroid | rs221783 | chr7_100695291_T_C_b38 | *GIGYF1* | 1.14x10^-26^ | 0.37 | 0.03 | rs221798 | 1.00 |

## Supplementary Table 30: Conditional analysis of common variant and *GIGYF1* pLOF

Conditional analysis was performed in individuals from the European ancestry population with array genotypes, exome sequencing data and biomarker measurements available.

| **phenotype** | **Variants(s)** | **adjustment** | **pvalue** | **Beta (SD)** |
| --- | --- | --- | --- | --- |
| glucose | *GIGYF1* pLOF | adj rs221783 | 5.29E-12 | 0.71 |
| glucose | *GIGYF1* pLOF | unadjusted | 5.66E-12 | 0.71 |
| HbA1c | *GIGYF1* pLOF | adj rs221783 | 6.38E-12 | 0.65 |
| HbA1c | *GIGYF1* pLOF | unadjusted | 6.83E-12 | 0.65 |
| glucose | rs221783 | adj pLOF | 1.41E-10 | -0.03 |
| glucose | rs221783 | unadjusted | 1.51E-10 | -0.03 |
| HbA1c | rs221783 | adj pLOF | 9.26E-07 | -0.02 |
| HbA1c | rs221783 | unadjusted | 9.91E-07 | -0.02 |

## Supplementary Table 31: Replication of rs221783 associations and meta-analysis

| **dataset** | **phenotype** | **chr** | **pos (hg19/hg38)** | **Ref**  **(effect allele)** | **Alt** | **rsid** | **MAF** | **pvalue** | **effect in SD or OR (95% CI)** |
| --- | --- | --- | --- | --- | --- | --- | --- | --- | --- |
| UKBB | Glucose | 7 | 100292914/  100695291 | T | C | rs221783 | 11.0% | 1.82x10^-11^ | -0.03  (-0.03, -0.02) |
| Biobank Japan | Glucose (blood sugar) | 7 | 100292914/  100695291 | T | C | rs221783 | 3.5% | 1.71x10^-4^ | -0.05  (-0.07, -0.02) |
| UKBB+Japan | Glucose | 7 | 100292914/  100695291 | T | C | rs221783 | - | 4.43x10^-14^ | -0.03  (-0.04, -0.02) |
| UKBB | T2D  (ICD10 E11) | 7 | 100292914/  100695291 | T | C | rs221783 | 11.0% | 0.005 | 0.96  (0.93, 0.99) |
| FinnGen R3 | T2D  (E4 DM2) | 7 | 100292914/  100695291 | T | C | rs221783 | 15.4% | 0.02 | 0.96  (0.93, 0.99) |
| UKBB+FinnGen | T2D | 7 | 100292914/  100695291 | T | C | rs221783 | - | 2.98x10^-4^ | 0.99  (0.94, 0.98) |

## Supplementary Table 32: Identification of causal genes at T2D GWAS loci

T2D and HbA1c associations for pLOF and missense variants in the two genes closest to T2D GWAS hits [1] were examined. Significant results are shown (p ≤ 2.41 x 10^-5^ adjusting for 2071 variant sets tested).

| **Variant set** | **title** | **pvalue** | **effect (beta/OR)** | **95% CI -** | **95% CI +** |
| --- | --- | --- | --- | --- | --- |
| ANK1 missense | HbA1c | 3.12E-19 | -0.13 | -0.16 | -0.10 |
| GCK missense | HbA1c | 1.86E-17 | 0.56 | 0.43 | 0.69 |
| GCK pLOF | HbA1c | 2.64E-17 | 1.29 | 0.99 | 1.59 |
| HNF1A pLOF | HbA1c | 2.14E-07 | 0.59 | 0.37 | 0.82 |
| TNRC6B pLOF | HbA1c | 2.36E-07 | 0.58 | 0.36 | 0.80 |
| CFTR pLOF | HbA1c | 1.4E-06 | -0.10 | -0.15 | -0.06 |
| SLC30A8 missense | HbA1c | 6.16E-06 | -0.18 | -0.26 | -0.10 |
| NF1 pLOF | HbA1c | 1.27E-05 | -0.25 | -0.37 | -0.14 |
| SLC30A8 pLOF | HbA1c | 1.44E-05 | -0.28 | -0.41 | -0.15 |
| IRS2 pLOF | HbA1c | 1.58E-05 | 0.69 | 0.38 | 1.00 |
| GCK pLOF | T2D | 2.96E-15 | 14.16 | 7.33 | 27.34 |
| HNF1A pLOF | T2D | 1.23E-09 | 5.27 | 3.08 | 9.00 |
| GCK missense | T2D | 1.7E-08 | 2.96 | 2.03 | 4.32 |
| TNRC6B pLOF | T2D | 2.00E-07 | 4.44 | 2.53 | 7.79 |
| IRS2 pLOF | T2D | 9.45E-06 | 5.88 | 2.69 | 12.89 |
| HNF4A missense | T2D | 1.01E-05 | 1.52 | 1.26 | 1.84 |

# Supplementary Note

We examined the quality and location of individual variants in the following sets; *GIGYF1* pLOF, *TNRC6B* pLOF and *PFAS* damaging missense variants.

For *GIGYF1*, all pLOF variants overlapped both *GIGYF1* protein-coding transcripts. The most common *GIGYF1* pLOF variant in our data (rs770150936, hg38 7:100687545:CA:C, MAF = 0.0054%), carried by 39 people, is predicted to result in a frameshift and premature termination of translation at position 234 (ENSP00000275732.4:p.Leu111ArgfsTer234). We examined quality metrics for this variant and found that it was sequenced to a mean depth of 33x and had an allele quality score of 1142 (in 70th percentile for quality). The 7:100687545:CA:C variant is present in gnomAD v2.1.1 and v3 and has a frequency in the non-Finnish European population of 0.0048% and 0.0031% respectively.

For *PFAS*, the missense variants contributing most to the association with HbA1c are hg38 17:8258163:G:A (rs141777945, MAF = 0.2%) resulting in a glutamate to lysine substitution at position 434 (ENSP00000313490.6:p.Glu434Lys) and hg38 17:8262979:G:A (rs34939404, MAF = 0.7%) resulting in an alanine to threonine substitution at position 466 (ENSP00000313490.6:p.Ala466Thr). These variants were sequenced to a mean depth of 31x and 24x respectively and both had quality scores in the 75^th^ percentile. Both are present at similar frequencies in gnomAD. These missense variants affect the longest protein-coding *PFAS* transcript ENST00000314666 (canonical and MANE select transcript).

Individual *TNRC6B* pLOF variants tended to be rare with the most common variants carried by just 9 (hg38 22:40270205:G:T; NP_001020014.1:p.Glu217Ter) and 7 (hg38 22:40281161:G:T NP_001155973.1:p.Glu1152Ter) individuals in European ancestry population respectively. This is consistent with the fact that the gene is constrained (pLI=1 and LOEUF=0.17). Both variants were sequenced to a mean depth of at least 42x and had high quality scores. Only 22:40281161:G:T is present in gnomAD and is flagged by the LOFTEE algorithm as potentially occurring in a misannotated exon (PHYLOCSF_UNLIKELY_ORF). Strikingly, 48 out of 71 carriers tested carried singleton variants. The large number of singletons together with the fact that *TNR6CB* is constrained suggest that some of these pLOF variants may not result in true loss of function or be due to sequencing errors.

# Regeneron Genetics Center Banner Author List and Contribution Statements ^1^

^1^ Affiliation: Regeneron Genetics Center, Regeneron Pharmaceuticals, Tarrytown, NY

All authors/contributors are listed in alphabetical order.

**RGC Management and Leadership Team**

Goncalo Abecasis, Aris Baras, Michael Cantor, Giovanni Coppola, Aris Economides, Luca A. Lotta, John D. Overton, Jeffrey G. Reid, Alan Shuldiner, Katia Karalis, Andrew Deubler and Katherine Siminovitch .

Contribution: All authors contributed to securing funding, study design and oversight. All authors reviewed the final version of the manuscript.

**Sequencing and Lab Operations**

Christina Beechert, Caitlin Forsythe, Erin D. Fuller, Zhenhua Gu, Michael Lattari, Alexander Lopez, John D. Overton, Thomas D. Schleicher, Maria Sotiropoulos Padilla, Louis Widom, Sarah E. Wolf, Manasi Pradhan, Kia Manoochehri, Ricardo H. Ulloa.

Contribution: C.B., C.F., A.L., and J.D.O. performed and are responsible for sample genotyping. C.B, C.F., E.D.F., M.L., M.S.P., L.W., S.E.W., A.L., and J.D.O. performed and are responsible for exome sequencing. T.D.S., Z.G., A.L., and J.D.O. conceived and are responsible for laboratory automation. M.S.P., K.M., R.U., and J.D.O are responsible for sample tracking and the library information management system.

**Genome Informatics**

Xiaodong Bai, Suganthi Balasubramanian, Andrew Blumenfeld, Boris Boutkov, Gisu Eom, Lukas Habegger, Alicia Hawes, Shareef Khalid, Olga Krasheninina, Rouel Lanche, Adam J. Mansfield, Evan K. Maxwell, Mona Nafde, Sean O’Keeffe, Max Orelus, Razvan Panea, Tommy Polanco, Ayesha Rasool, Jeffrey G. Reid, William Salerno, Jeffrey C. Staples,

Contribution: X.B., A.H., O.K., A.M., S.O., R.P., T.P., A.R., W.S. and J.G.R. performed and are responsible for the compute logistics, analysis and infrastructure needed to produce exome and genotype data. G.E., M.O., M.N. and J.G.R. provided compute infrastructure development and operational support. S.B., S.K., and J.G.R. provide variant and gene annotations and their functional interpretation of variants. E.M., J.S., R.L., B.B., A.B., L.H., J.G.R. conceived and are responsible for creating, developing, and deploying analysis platforms and computational methods for analyzing genomic data.

**Clinical Informatics**

Michael Cantor and Dadong Li

Contribution: All authors contributed to developing the phenotype definitions.

**Translational and Analytical Genetics:**

Adam Locke, Niek Verweij, Jonas Nielsen, Jonas Bovijn, Tanima De, Mary Haas, Parsa Akbari and Olukayode Sosina

Contribution: All authors contributed to the analytical analysis of the project.

**Research Program Management**

Marcus B. Jones, Jason Mighty, Michelle G. LeBlanc, and Lyndon J. Mitnaul,

Contribution: All authors contributed to the management and coordination of all research activities, planning and execution. All authors contributed to the review process for the final version of the manuscript.

# DiscoverEHR Collaboration Personnel

**Regeneron personnel ^1^:**

Goncalo Abecasis, Aris Baras, Michael Cantor, Giovanni Coppola, Aris Economides, Luca A. Lotta, John D. Overton, Jeffrey G. Reid, Alan Shuldiner, Andrew Deubler, Katia Karalis, Christina Beechert, Caitlin Forsythe, Erin D. Fuller, Zhenhua Gu, Michael Lattari, Alexander Lopez, Thomas D. Schleicher, Maria Sotiropoulos Padilla, Karina Toledo, Louis Widom, Sarah E. Wolf, Manasi Pradhan, Kia Manoochehri, Ricardo H. Ulloa, Xiaodong Bai, Suganthi Balasubramanian, Leland Barnard, Andrew Blumenfeld, Gisu Eom, Lukas Habegger, Alicia Hawes, Shareef Khalid, Evan K. Maxwell, William Salerno, Jeffrey C. Staples, Ashish Yadav, Dadong Li, Marcus B. Jones, Lyndon J. Mitnaul, Jason Mighty, Andrew Deubler, Katia Karalis, Katherine Siminovitch

^1^ Affiliation: Regeneron Genetics Center, Regeneron Pharmaceuticals, Tarrytown, NY

**Geisinger personnel ^2^:**

Lance J. Adams, Jackie Blank, Dale Bodian, Derek Boris, Adam Buchanan, David J. Carey, Ryan D. Colonie, F. Daniel Davis, Dustin N. Hartzel, Melissa Kelly, H. Lester Kirchner, Joseph B. Leader, David H. Ledbetter, Ph.D., J. Neil Manus, Christa L. Martin, Raghu P. Metpally, Michelle Meyer, Tooraj Mirshahi, Matthew Oetjens, Thomas Nate Person, Christopher Still, Natasha Strande, Amy Sturm, Jen Wagner, Marc Williams

^2^ Affiliation: Geisinger Health System, Danville, PA

References

1. Vujkovic M, Keaton JM, Lynch JA, Miller DR, Zhou J, Tcheandjieu C, et al. Discovery of 318 new risk loci for type 2 diabetes and related vascular outcomes among 1.4 million participants in a multi-ancestry meta-analysis. Nat Genet. 2020;52(7):680-91. Epub 2020/06/17. doi: 10.1038/s41588-020-0637-y. PubMed PMID: 32541925; PubMed Central PMCID: PMCPMC7343592.

2. McKusick-Nathans Institute of Genetic Medicine JHUB, MD). Online Mendelian Inheritance in Man, OMIM. <https://omimorg/>.

3. Flannick J, Mercader JM, Fuchsberger C, Udler MS, Mahajan A, Wessel J, et al. Exome sequencing of 20,791 cases of type 2 diabetes and 24,440 controls. Nature. 2019;570(7759):71-6. Epub 2019/05/24. doi: 10.1038/s41586-019-1231-2. PubMed PMID: 31118516; PubMed Central PMCID: PMCPMC6699738.
